# A global, proxy-based assessment of building climatization vulnerability

**DOI:** 10.1038/s41598-026-58888-y

**Published:** 2026-07-28

**Authors:** P. Florio, J. H. Uhl, P. Politis, M. Melchiorri, C. Maduta, K. Krasnodębska, A. M. Martinez

**Affiliations:** 1 European Commission, Joint Research Centre (JRC), Ispra, Italy; 2 Stanisław Leszczycki Institute of Geography and Spatial Organization, Polish Academy of Sciences, Warsaw, Poland; 3 European Dynamics Belgium S.A., Brussels, Belgium

**Keywords:** Climate sciences, Environmental sciences, Environmental social sciences, Geography, Geography

## Abstract

Building compactness and construction epoch are relevant proxies of energy efficiency in buildings. By integrating building stock and Earth observation data, we characterize buildings globally and identify vulnerable areas dominated by old building age and unsuitable compactness in relation to climate conditions. Such areas are home to a population of 48 million persons that could increase by 10% to 30% by 2100 under likely socioeconomic pathways, shifting from cold regions of Eurasia to the heat-prone Sahel zone, Gulf countries and Pakistan. Results reveal stark disparities in building energy efficiency across income levels, urbanization patterns, and climate zones. Almost half of buildings globally (i.e., 43%) were built before 1980, when few to no energy efficiency measures existed, and these buildings are concentrated mainly in temperate and cold-climate urban areas in high-income countries. Low-income countries experienced more recent, lightweight and less compact urban expansion, which exposes them to the forecasted climate warming: this could increase their energy demand, hinder climate adaptation and imposes further challenges to ensure affordable thermal comfort for all.

**Supplementary Information:**

The online version contains supplementary material available at 10.1038/s41598-026-58888-y.

## Introduction

### The relevance of energy efficiency in buildings and its uptake

Globally, buildings account for 30% of global final energy consumption and 28% of global energy-related emissions^1,2^: despite increasingly binding energy efficiency targets^3^ and new energy efficiency measures and technologies^4^, building energy demand grew by over 20% between 2000 and 2017^2^. Space heating is currently the dominant end-use in building energy consumption, but research suggests that while heating demand may stabilize or decrease^5,6^, cooling needs are expected to increase 2–3 times in non-residential buildings, and 3–20 times in residential buildings by 2050^7^, with significant regional variations. This trend implies a decrease of global CO_2_ emissions for space heating by 34%−52%, and an increase by 58%−85% for space cooling by 2050^6^. With over 80% of the global economic energy efficiency potential in buildings remaining untapped^8^, identifying worst energy-performing buildings to prioritize in a renovation scenario becomes essential.

Locating and quantifying such buildings at global scale is challenging. Crucial areas are countries lacking building energy regulations and standards, as around 70% of building floor area additions to 2050 will occur in places with limited building energy codes in place today, if any^2^. The inhomogeneous definitions and methods to assess energy efficiency in buildings across countries is starkened by the scarcity of necessary data, with many regions of the world lacking even most essential building registers.

In this context, emerging new datasets rely on Earth observation and aerial imagery to map buildings and to estimate their characteristics in a homogeneous way, with nearly seamless global coverage. Proxy indicators, such as building compactness and construction epoch, can provide an estimate of energy efficiency supporting policy actions. These indicators can be further refined using local-specific analyses at regional scale, enabling researchers to quantify and assess carbon emissions from building operation across cities^9^.

### Building compactness and construction epochs are key estimators of building energy efficiency

Building compactness^10^ and construction epoch^11^ are among the most relevant proxies of energy efficiency in buildings^12^, along with building usage type (mainly residential or non-residential), the proportion of glazing and orientation relative to cardinal directions^13^.

**Fig. 1:**
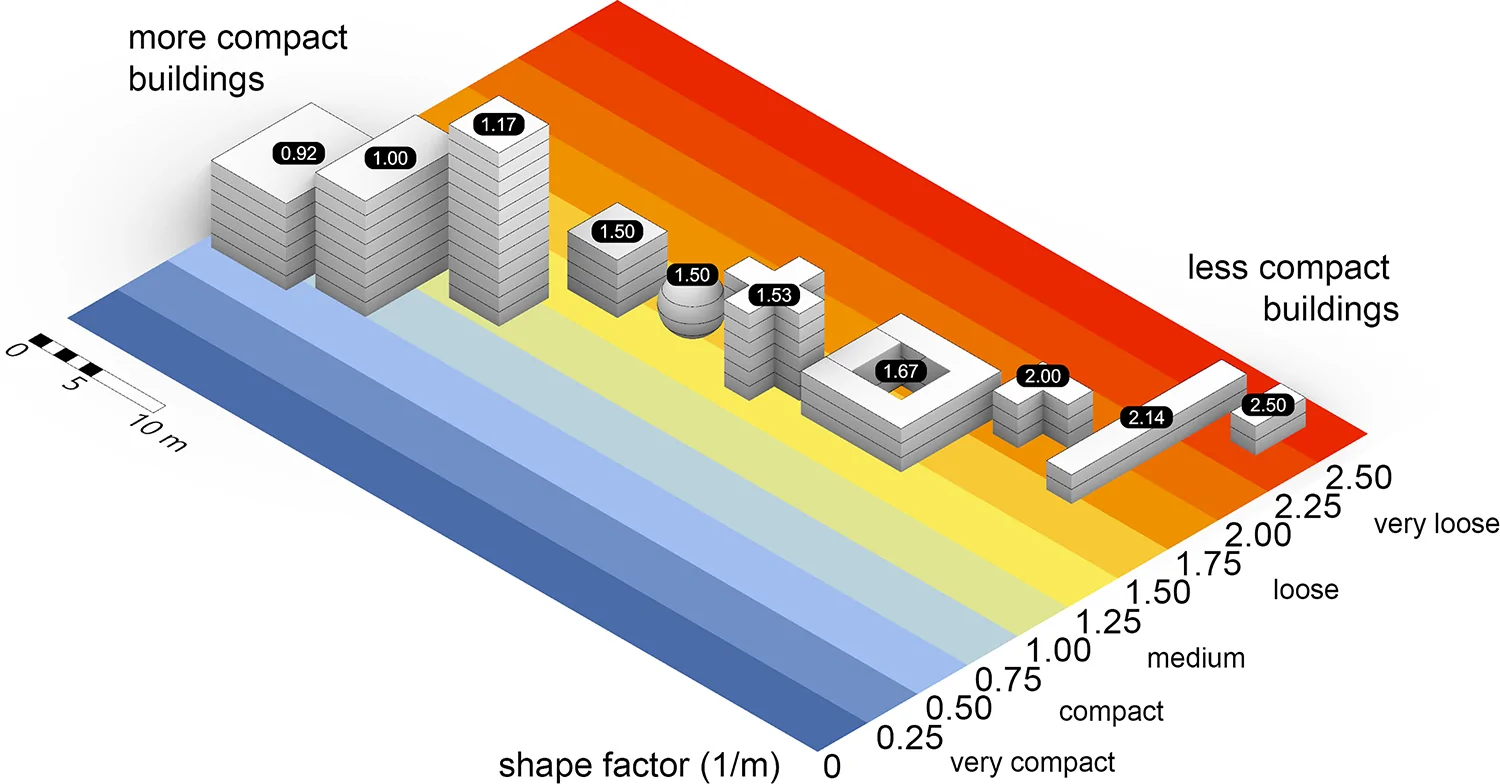
Schematic representation of the relation between the building shape factor and compactness in various building archetypes with different sizes and shapes.

The compactness of a building can be measured by the shape factor (Eq. 1), which is defined as the ratio of outdoor exposed building surface area (S) to heated volume (V). Expressed in units of m^2^/m^3^ (1/m), the shape factor is inversely proportional to the size of the building, with higher values indicating a less compact structure, and lower values a more compact one (Fig. 1). The building shape factor has been widely recognized as a critical factor in determining a building’s energy efficiency^14–18^, interwoven with energy consumption and environmental sustainability.

The building construction epoch has been employed as an estimator of energy efficiency^19^, especially in residential buildings, through the use of building typologies that associate the construction practice with overall energy performance^20^. The earliest efforts in building energy efficiency date back to the energy crises of the 1970 s, which spurred a focus on energy conservation and demand optimization^21,22^. Climate change policies have intensified the focus on building energy efficiency, especially in high-income countries, where the past decade has seen a widespread adoption of zero-energy building concepts. Dating the construction of a building is essential for large-scale building energy demand estimations, and the use of building construction epoch as predictor for energy demand using Machine Learning techniques has promising potential^23,24^.

### Building energy efficiency and “climatization vulnerability”

We aim to address the gap in understanding global building energy efficiency. We provide a method to identify buildings at risk of higher heating and cooling needs and experiencing thermal discomfort, with the use of open data derived from Earth observation data. By exploring the complex relationships between building compactness, construction epoch, and energy efficiency in relation to climate, this study introduces a novel framework for spatially comparative and multidimensional monitoring of energy efficiency drivers of the built environment, encompassing different regions of the world, across multiple climate zones, socio-economic realities and the rural-urban gradient, with a focus on the most vulnerable areas. Herein, the concept of “climatization vulnerability” is -defined as the strain on energy demand imposed by old building age in combination with unsuitable compactness, in relation to climate. This indicator serves as a tool to identify climate-vulnerable buildings, enabling policy makers, governmental authorities and researchers to explore options to reduce the risk of thermal discomfort, address energy poverty, and achieve greater energy and emission savings.

## Results

**Fig. 2:**
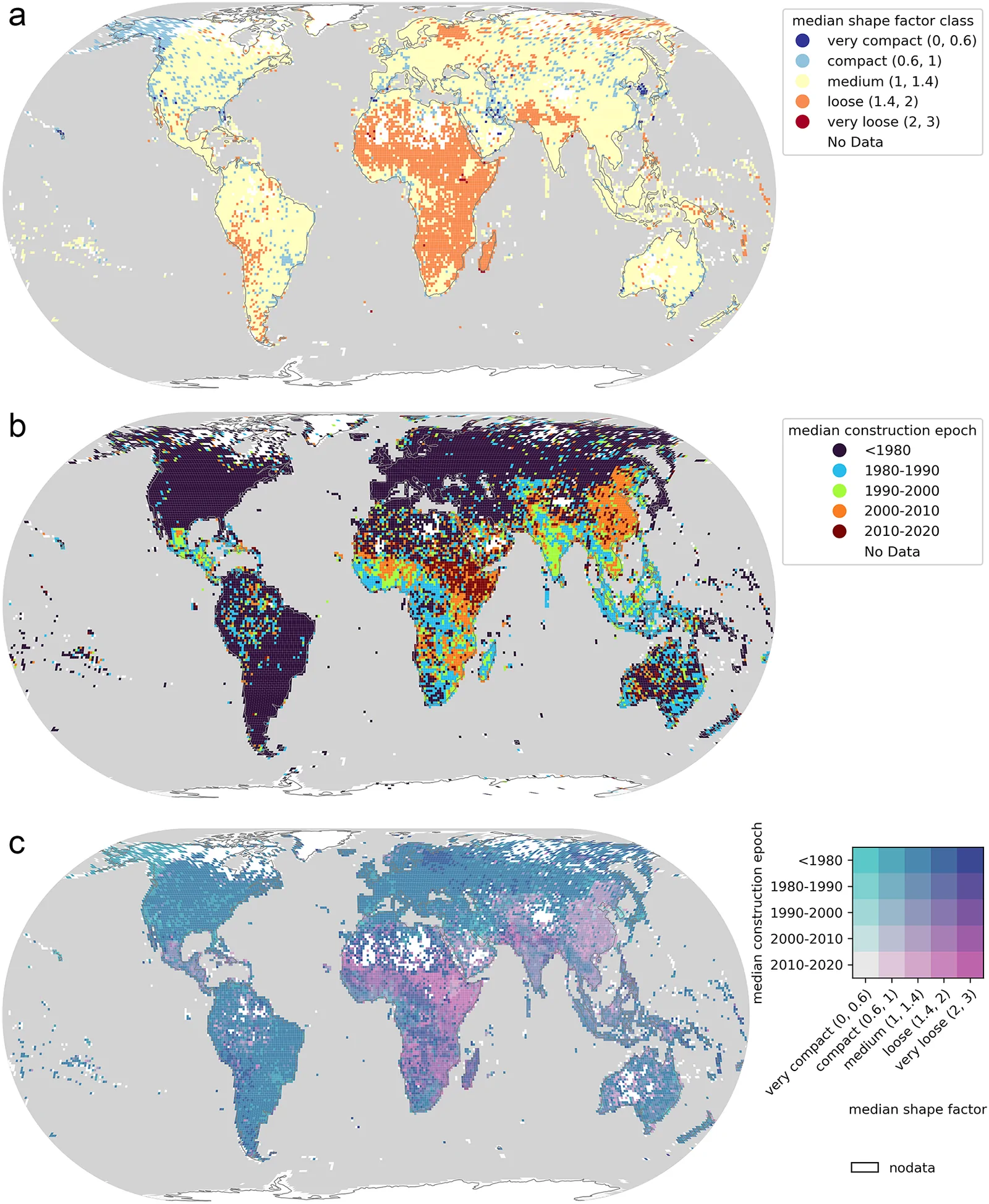
Shape factor and construction epoch aggregates in 100 km tiles, derived from GHS-OBAT building footprint data^25^, integrated with the East Asia building footprint dataset ^26^. (**a**): median shape factor representing building compactness, (**b**): median building construction epoch, (**c**): bivariate map of the two components. Maps created with Cartopy, with free vector and raster basemap data from Natural Earth @ naturalearthdata.com.

### Buildings are more compact in Eastern Asia and looser in Africa

The maps in Fig. 2 highlight compactness and construction epoch extremes, subdividing the globe into 100 × 100 km tiles. Loose and very loose (i.e., least compact) buildings are found in Sub-Saharan Africa, south Asia, Latin America and Middle East (Fig. 2a). Overall, more compact buildings (i.e., a shape factor lower than 1) find place in South Korea, the Persian Gulf, Japan, Alaska (U.S.A.), and to a lower extent in the rest of the U.S.A., the region of Sao Paulo (Brazil), and continental Europe.

According to Fig. 2b, older buildings are mainly located in Europe, North America, and former Soviet countries with some pockets in Latin America, the Middle East and East Asia. Most of the building construction after 1980 took place in West Africa, South-Eastern Asia and large parts of Australia (1980–1990), Central America and India (1990–2000), China and East Africa (2000–2010), with the most recent construction epoch dominating in the Horn of Africa (2010–2020).

Figure 2c shows few areas with older, looser buildings, which are particularly vulnerable to high energy consumption for heating and cooling. Very old (i.e., built before 1980) and loose buildings (i.e., shape factor higher than 1.4) are concentrated in the Baltic Region of Russia and more sparsely distributed over the rest of Russia, Northern Africa and the Sahel, as well as the Andean region. More recently built, loose buildings are predominant in the remaining African regions, Northern India and Bangladesh.

This geography highlights two contrasting realities. On one hand, it shows how ongoing urban development and recent, fast urbanization have led to less compact settlements in Africa and India, unlike China. On the other hand, it reveals that Europe and North America result in more established and consolidated cities.

### Compactness has a distinct urban-rural gradient

**Fig. 3:**
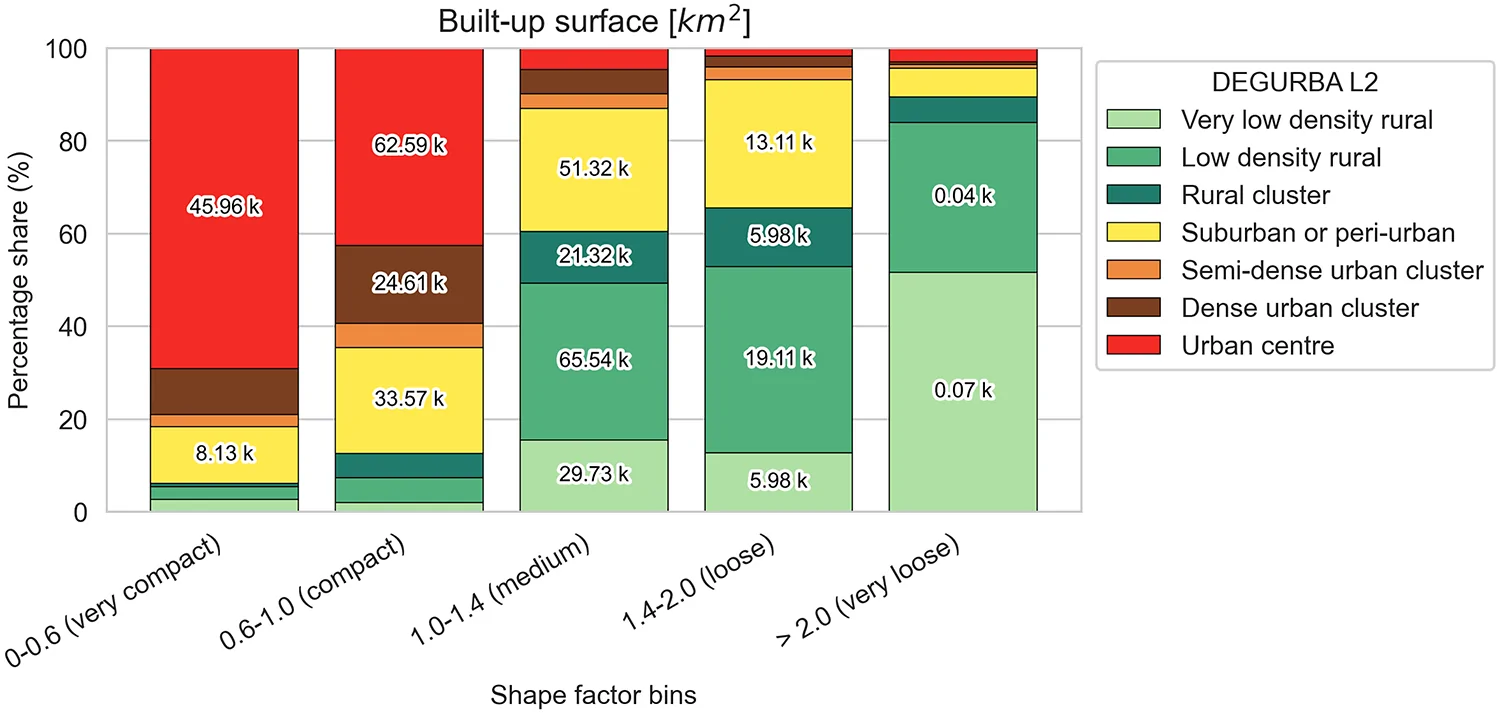
Share of global built-up surface by bins of shape factor and by Degree of Urbanization level 2 classification at grid level (GHS-SMOD) for 2025^52,54^. Bars are labelled with surface values in km^2^.

Most compact buildings worldwide are found in urban centers, the densest settlement category, which hosts almost 75% of their footprint surface overall (Fig. 3). Increasingly looser buildings find place in suburban, peri-urban and rural areas, with more than 85% of overall medium, loose and very-loose built-up surface (shape factor higher than 1). Consequently, more than 90% of built-up surface in urban centers globally is compact (shape factor lower than 1), in contrast to only 10% in very low/low density rural areas. Suburbanization and urban sprawl towards rural areas favor more relaxed building morphologies, characterized by low-rise buildings and lower building compactness.

### New buildings in urban centers became less compact throughout decades but follow different trajectories across world regions

**Fig. 4:**
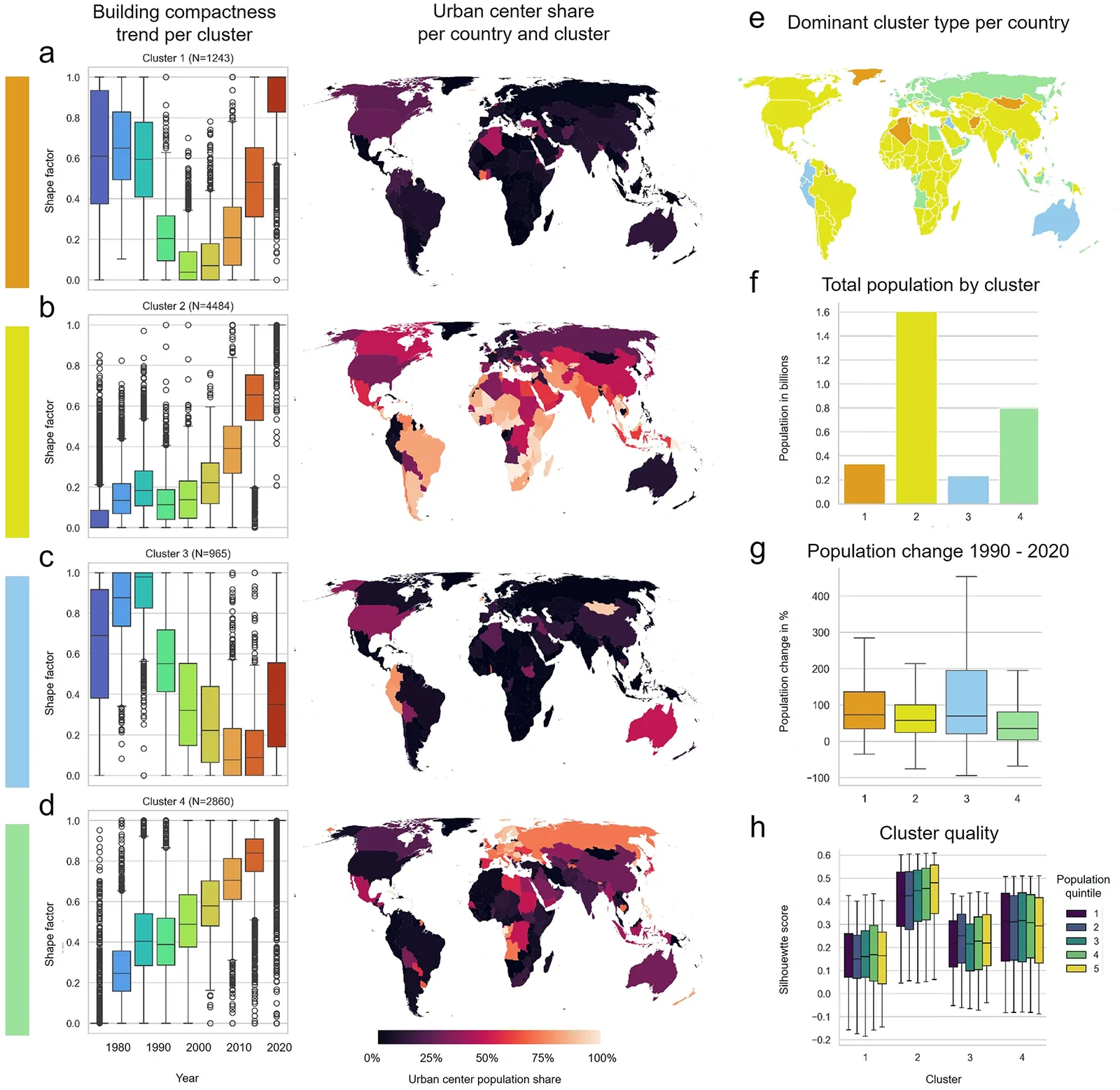
Results of a city-level time series cluster analysis of building compactness using building compactness and construction epoch data for over 9,000 urban centers. Panels (**a**) - (**d**) show the compactness trends per cluster, measured in relative terms for each urban center, along with their corresponding geographic distributions. Panel (**e**) shows the dominant cluster type per country, while panel (**f**) shows the total population residing in urban centers of the four clusters, and the distributions of relative urban population change from 1990 to 2020 are shown in (**g**). Panel (**h**) shows distributions of the Silhouette score for each urban center, stratified by cluster and population quintile. Higher Silhouette scores indicate that an urban center is highly similar to other urban centers in the same cluster, and very dissimilar to urban centers assigned to other clusters. Maps created with Matplotlib, with free vector and raster basemap data from Natural Earth @ naturalearthdata.com.

A global analysis of over 9,000 urban centers reveals a city-level relationship between building construction epoch and compactness, characterized by four distinct clusters. Our results show that, within urban centers, newer buildings tend to be less compact than older ones, as reflected in an increasing average shape factor in three clusters (Fig. 4a, b and d). Regional patterns are prominent, indicating homogeneous urbanization trends across neighboring countries, coupling building compactness and construction epoch at the city level.

Two dominant clusters emerge (Fig. 4): cluster 2, characterized by a recent, sharp increase in shape factor, indicating a decreasing compactness in recent decades, is concentrated in the Global South (Fig. 4b) and represents the most common building age - compactness relationship (Fig. 4 e,f); cluster 4, showing a linear increase in shape factor over time, is concentrated in Eurasia (Fig. 4d). North America, which typically exhibits suburbanization patterns beyond urban center boundaries, is divided among clusters 1, 2, and 3. Cluster 3, marked by a decrease in shape factor over time, reveals a more compact construction in Andean countries and comprises cities with a rapid population growth after 1990 (Fig. 4g); cluster 3 may also be interpreted as matching the trajectory of cluster 1, with a time lag of 10–20 years. See Supplementary Fig. S9 for country-level cluster statistics and Supplementary Fig. S10 for example cities.

Cluster distinctiveness, measured by the Silhouette score (Fig. 4h), is highest for cluster 2, indicating high similarity among its urban centers and dissimilarity with others. Large urban centers are more representative of cluster 2, while small and medium-sized centers are more typical of clusters 1, 3, and 4, as shown by the decrease in average Silhouette score by population quintile (Fig. 4h).

### Similar building age - compactness relationships across cities indicate similar socioeconomic settings

**Fig. 5:**
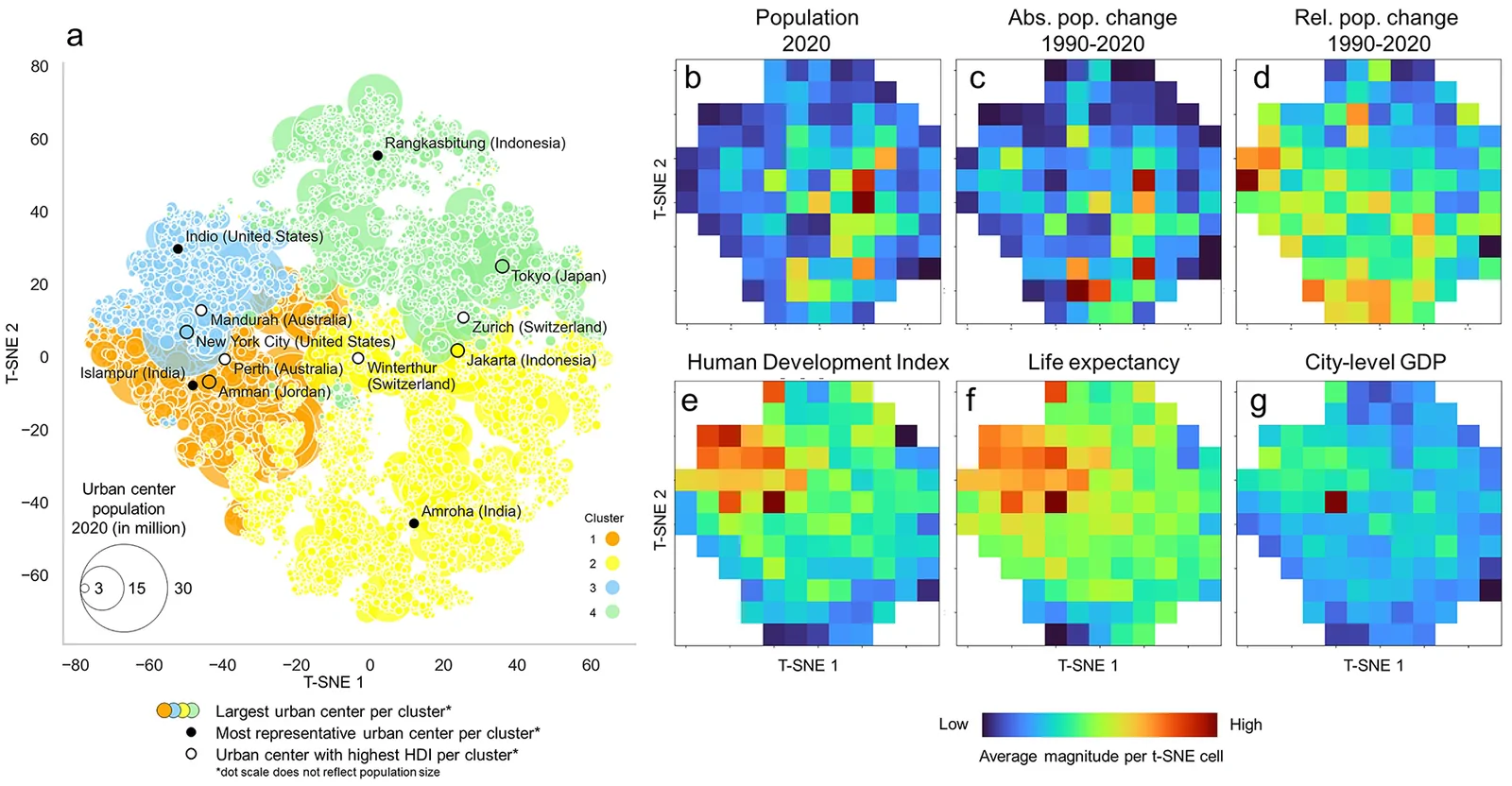
Arrangement of over 9,000 urban centers in a 2D-similarity space, based on a t-SNE transform of their compactness-age trends and visualization of various attributes, including (**a**) cluster membership, highlighting selected urban centers per cluster, (**b**) average urban center population in 2020 and (**c**) absolute and (**d**) relative population change 1990–2020, averaged across urban centers per cell. The bottom row shows city-level socio-economic attributes including (**e**) Human development index (HDI), (**f**) Average life expectancy, (**g**) city-level GDP.

Similar city-level building compactness trajectories across construction epochs can be visualized in a similarity space derived from dimensionality reduction, where proximity of urban centers indicates trajectory similarity (Fig. 5a). The results reveal that city attributes, including population size (Fig. 5b), population growth (Fig. 5c), human development indicators (Fig. 5e, f), and economic factors (Fig. 5g), exhibit distinct spatial patterns in the compactness trajectory similarity space. This suggests that cities with similar building compactness trajectories over time, as measured by city-level time series, tend to share similar size-related and socio-economic characteristics, with regional concentrations confirming those observed in Fig. 4 (see also Supplementary Fig. S. 12).

### Country income marks disparity in building features linked with energy efficiency

**Fig. 6:**
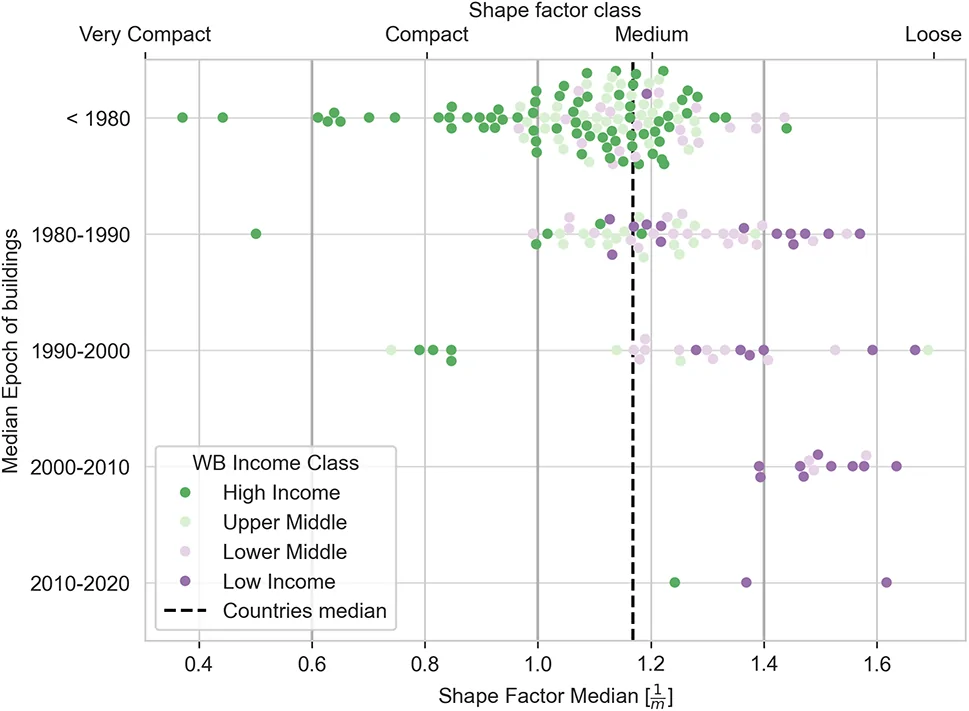
Swarm plot of median shape factor and construction epoch by country in the world and by World Bank income class.

Income levels relate with geographic patterns of building compactness and construction epoch. This evidence emerges as well by using the World Bank classification FY24^27,28^, which categorizes countries by income level. As shown in Fig. 6, lower and lower-middle-income countries have experienced more recent urbanization, with most of their buildings constructed after 1980. However, the efficiency of newer buildings in these countries is not guaranteed, as only approximately 25% of lower-middle and low-income countries implement building energy codes, compared to around 50% of upper-middle and high-income countries together^29^.

In addition, buildings in low-income countries are less compact on average (i.e. higher shape factors), and more lightweight, as revealed by recent research^30^. The combination of low thermal mass and low compactness makes these buildings more susceptible to daily temperature fluctuations without adequate climate control.

**Fig. 7:**
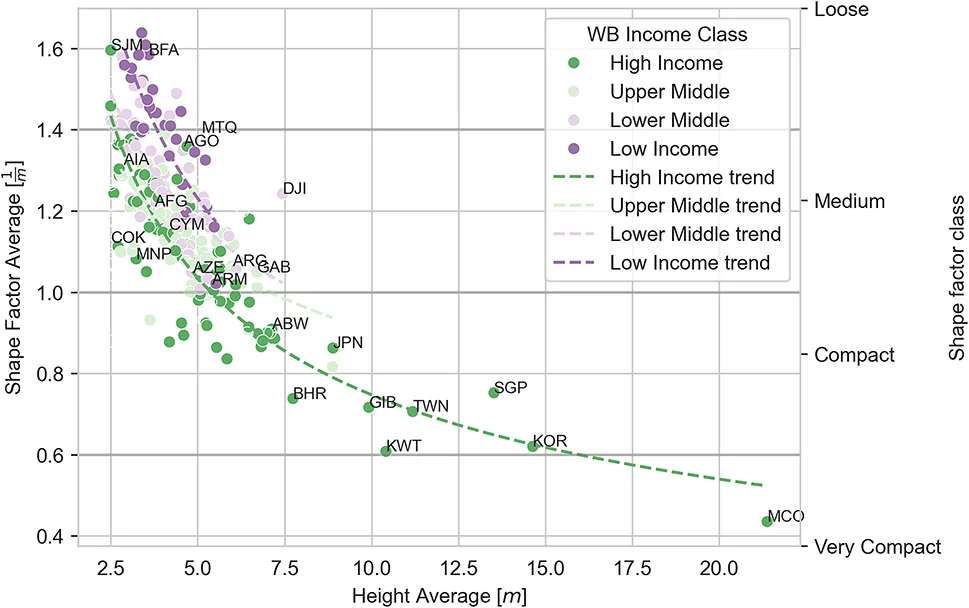
Scatterplot of average height and shape factor by country in the world and by World Bank income class. Logarithmic trendlines by income class are dashed.

High-rise buildings are characterized by increased building compactness, as the conditioned volume is more concentrated, resulting in a lower shape factor (see Eq. 1 in Materials and Methods section below). However, for similar building heights, high-income countries tend to have higher compactness (lower shape factors, as seen in Fig. 7). This suggests that high-income countries make more efficient use of land^31^, as they would have larger footprints for the same perimeter length (see Eq. 1).

The inefficiency in building compactness, thermal mass, and land use, combined with an already high energy intensity per unit of GDP (which is approximately 50% higher in low-income compared to high-income countries)^32^, puts a significant strain on low-income countries. Low- and middle-income countries, excluding China, are projected to experience a nearly 30% increase in energy demand over the next decade, with approximately three-quarters of this demand being met by fossil fuels, making it imperative that these countries prioritize energy efficiency in buildings^8^.

### Inequalities are intensified in low-income areas

**Fig. 8:**
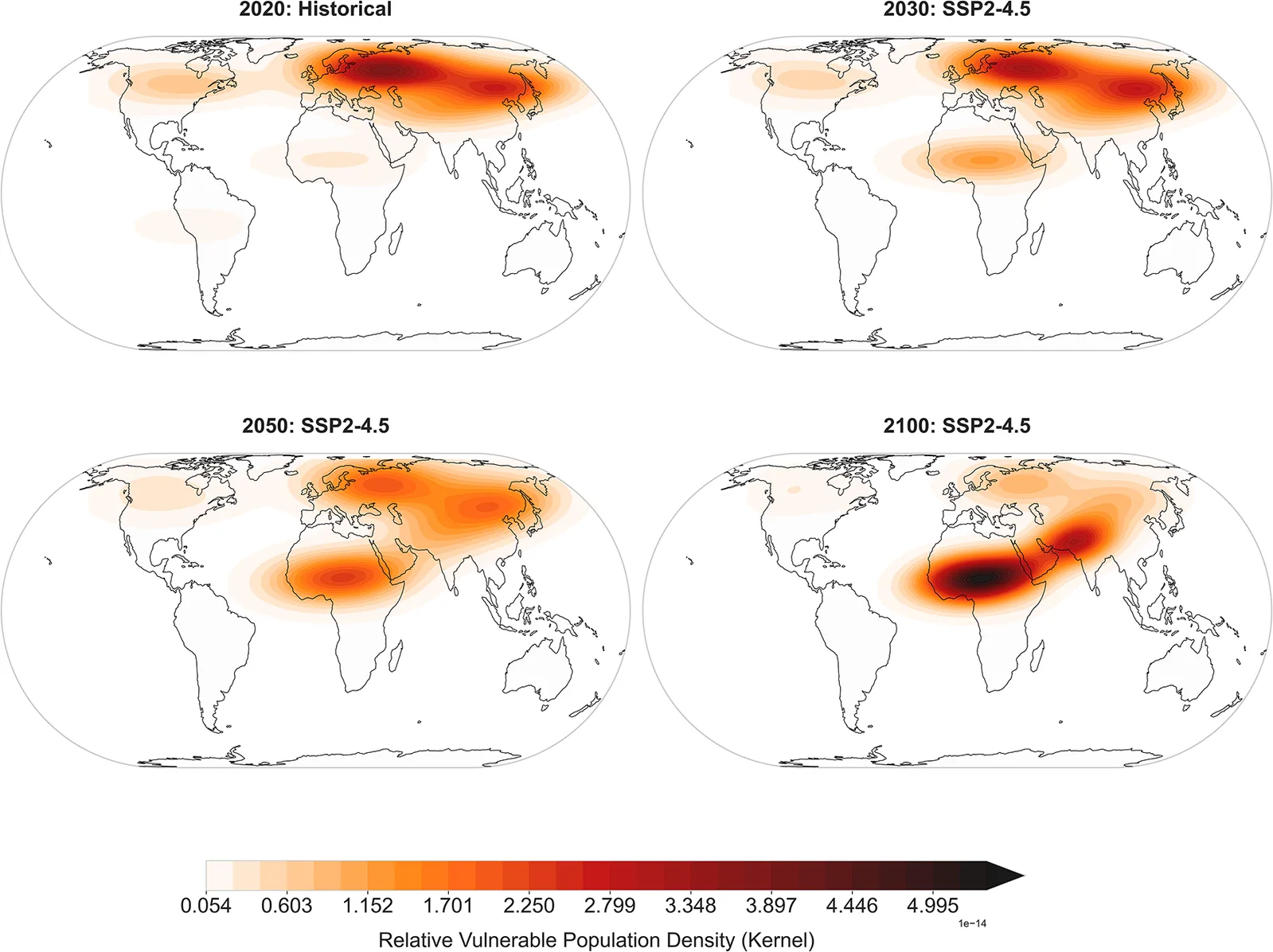
Relative vulnerable population density (gaussian kernel density) in 2020, and in 2030, 2050, 2100 (according to SSP 2–4.5), resulting from old construction epoch and unsuitable shape factor in relation to the local heating and cooling degree days, per 100 × 100 km tiles. Maps created with Cartopy, with free vector and raster basemap data from Natural Earth @ naturalearthdata.com.

Climate plays a crucial role in the spatial distribution of building compactness (see Supplementary Sect. 5), as construction typologies and building forms are closely tied to the local climatic context. Low compactness (high shape factor) is generally associated with reduced climatization efficiency, as a larger surface area is exposed to the outdoors for a given conditioned volume (see Materials and Methods section for details). However, extremely high compactness can hinder natural ventilation and passive cooling in hot, humid climates and reduce the benefits of solar gains in colder climates. The combination of an old construction epoch, implying outdated energy efficiency standards, and building compactness that is ill-suited to the climate can lead to increased reliance on active climatization, resulting in higher energy demand. This *vulnerability* is independent of envelope thermal insulation and the presence or efficiency of heating and air conditioning systems, of which little is known at this scale. Consequently, buildings in these *vulnerable* areas are likely more susceptible to climate-related stress, and occupants may experience thermal discomfort in the absence of air conditioning.

**Fig. 9:**
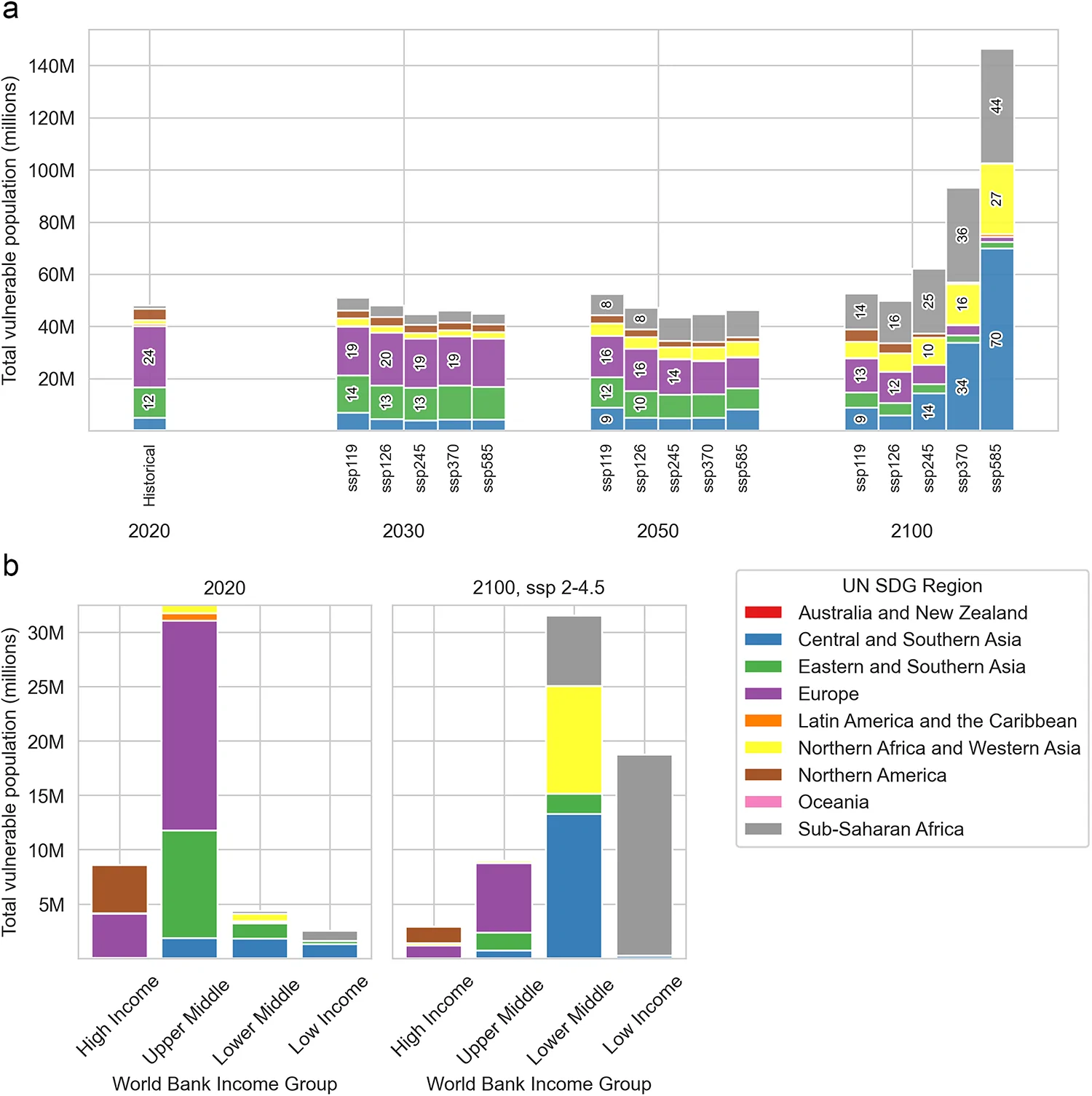
(**a**): Evolution trends of vulnerable global population in 2030, 2050 and 2100, by SSP scenarios, (**b**): Share of vulnerable global population in 2020 vs. 2100 (SSP 2–4.5), split by World Bank income class and UN SDG region.

The vulnerability criteria rely on old construction epoch and high shape factor limits, the latter with a threshold anchored to local heating and cooling degree days (details provided in the Materials and Methods section). Figure 8 shows the global density of vulnerable population: rural areas of the cold region (Europe, Eastern Asia, Northern America) stand out in 2020, by effect of isolated, low-rise, loose building clusters in sparse settlements. Global warming will mitigate climate in this region, relieving 40% − 90% of vulnerable population by 2100, depending on the shared socioeconomic pathway - SSP scenario. However, climate change shifts the impacts towards the equator, where cooling degree days will increase by at least 75%, but could become up to 5 times higher by 2100, with noticeable effects already in 2030.

The stress on tropical and tropicalized countries is expected to raise; from population estimates of GHS-WUP^33^, the global vulnerable population could remain stable at 48 million or increase up to 2–3 times by 2100, depending on the climate projections, primarily in Sub-Saharan Africa and Asia (Fig. 9a). These findings reveal a complex scenario for low-income countries (see Fig. 9b), where buildings tend to be less compact on average (Figs. 6 and 7), home to a large part of the global population. This disparity highlights existing social inequalities^34^, particularly across the urban-rural gradient.

## Discussion

We introduce the concept of “climatization vulnerability” and apply it to a global building dataset, using thresholds of building compactness and construction epoch (see Materials and Methods section). This framework highlights the importance of such building features in informing energy efficiency policy. While the selection of parameters and thresholds may be refined based on further evidence, our analysis reveals distinct scenarios.

The results demonstrate how country income, driving settlement expansion and habitat standards, is related to building construction epoch and compactness. The degree of urbanization and construction practices in relation to climate all impact building compactness. In turn, building characteristics such as compactness and construction epoch have an impact on energy efficiency and, ultimately, on climatization needs, leading to climate-related stress in some vulnerable areas.

Notably, our results show that the economic context of a country plays a significant role in shaping its climatization strategies. Low-income countries, where air conditioning is less affordable and the use of energy is less economically efficient, will experience dramatically higher heat stress already in the near future, burdening active climatization, implying higher energy demand and requiring more effort to adapt to climate change. In contrast, high-income countries, which benefit from an already well-regulated construction market and an efficient energy dispatch system, will be relieved by a milder climate induced by global warming, although still susceptible to heatwaves and peak demand. Dispersed rural areas with scattered buildings are more vulnerable in this case, with measures like thermal insulation of old buildings that can lead to an increasingly optimized and efficient use of energy in the building stock overall.

Globally, nearly half of buildings (43%) were constructed before 1980, when energy efficiency measures were scarce. These buildings are concentrated in temperate and cold urban areas of well-regulated Europe and North America, where compact buildings are predominantly found in urban areas and loose buildings in rural areas (Supplementary Fig. S. 2). In compact urban areas with heavyweight construction, replacing windows and installing efficient climatization systems can significantly reduce energy demand, without requiring complex renovation works. In contrast, rural areas with loose buildings may benefit from opaque envelope insulation and active solar energy production^35^. Careful assessment of energy efficiency upgrades is essential to prevent technological and infrastructural lock-ins that could hinder further energy and greenhouse gas emissions reduction, in line with national climate targets. Adaptation measures, such as reflective building materials, expansion of green spaces and permeable pavements should also be deployed to prevent overheating in the context of rising temperatures in these areas^36^.

In tropical and hot regions of Africa and Southern Asia, newer buildings constructed from lightweight materials dominate the landscape. Our results show that these buildings are increasingly loose across most recent construction epochs, both in urban and rural areas (Supplementary Figs. S. 2, S. 5). To mitigate high energy demand, building regulators should establish thermal mass standards to control thermal lag and manage excessive heat transfer through compactness limits and insulation guidelines^17^. Additionally, exceptionally compact buildings in dense settlements should be designed with large openings for ventilation, and urban planning should incorporate microclimatic mitigation and adaptation strategies, such as increased building spacing, façade shading, and vegetation infills^37,38^. This is essential to grant reasonable thermal comfort, especially in areas where air conditioning is less affordable, e.g. low-income countries.

Moreover, four types of building compactness trajectories emerge from the global set of urban centers, mostly characterized by compactness decrease from the old towards the new building stock. These trajectories exhibit regional patterns linked with income classes, suggesting that broader economic contexts influence urban development trends across global regions.

Importantly, our analysis and the underlying data have a few limitations. Compactness is computed at a level of detail that assumes all roofs are flat; however, ignoring roof shape only slightly affects the shape factor accuracy (see dedicated Supplementary Sect. 8), which depends largely on building height obtained from GHS-BUILT-H^39^, and on the quality of building footprint digitization, potentially affected by the misrepresentation of adjacent walls and courtyards.

Moreover, older buildings are not necessarily all energy inefficient, as little is known about their renovation status, presence of envelope insulation, energy production and conversion systems.

The International Energy Agency (IEA) estimates that worldwide building renovations occur at about 1% rate per year^40^, but many of these works do not achieve significant energy‑efficiency gains. In the European Union, the rate of deep‑renovations that meaningfully improve a building’s energy performance (i.e. energy savings of at least 60%) was estimated at roughly 0.2% per year in 2021^41^. If a 0.2% constant deep‑renovation rate were applied to pre‑1980 buildings in both the EU and the United States from 1980 to 2020, about 8% of such buildings would have had their energy efficiency upgraded. Since not all upgraded buildings are located in vulnerable areas, removing random buildings from the pre-1980 epoch would, on average, reduce the share of vulnerable populations in those territories by a comparable proportion, which can be rounded to circa 10% (Supplementary Table S. 2). Therefore, 10% of the vulnerable population in 2020 can be subtracted from such regions as a safe uncertainty margin. As for the future, annual building renovation rate remain uncertain, beyond the European Union target to at least double the rate by 2030 compared to the 2020 average annual rate of 1%^42^.

Generalized deductions from building construction epoch and compactness are limited to passive climatization strategies and not always valid, as they also do not consider occupants’ physiological adaptation to climate, culture and behavior. The estimated climatization needs cannot be attributed solely to building form and age, as analyzed in this study, but are also strongly influenced by climate‑responsive adaptation, occupant behavior, and the design of energy policies^43,44^. Climatization vulnerability is sensitive to assumptions about old buildings and reference values for threshold compactness (see sensitivity analysis in Supplementary Fig. S. 8); however, it is also influenced by socio-economic factors, including levels of deprivation, which, although outside the scope of this analysis, undoubtedly play a significant role^45^.

Although adapted for large scale aggregation and derived statistics, the building footprint dataset used for our analysis cannot withstand the accuracy of on-field assessments, both in terms of coverage and in terms of accuracy of certain attributes (see references in the Materials and Methods section and Supplementary Fig. S. 1 for details). However, the large-scale use of building vector footprints for global analytics remains a pioneering field of research, of which this study is a possible perspective.

The term “compactness” refers specifically to building design, distinct from settlement compactness^31^. While new developments may feature compact settlements characterized by an efficient layout of built land with optimized infrastructure in-fills^46^, buildings themselves often have less compact shapes, with the same living area distributed across more buildings.

## Materials and methods

### Data sources and integration

Key parameters such as built-up surface growth, dominant age of the built stock, building volume, building height, residential vs. non-residential use of buildings, and the classification of urban, suburban/peri-urban, and rural areas are derived from the Global Human Settlement Layer (GHSL) data. These elements are aligned to the explicit semantic definitions in GHSL for these abstractions and measurements, ensuring consistency and clarity^47^.

Building compactness (measured by the shape factor) and construction epoch are obtained from GHS-OBAT^48^, a dataset that attributes building footprint geometry features from Overture buildings (release 2024-07−22.0)^49^ with building height, shape factor, use and construction epoch attributes as columns, split by country and administrative areas. Building height, in particular, is estimated at the footprint-level using hectare-level estimates from GHS-BUILT-H^39^. To improve completeness in Eastern Asia, Overture buildings are complemented with an dedicated open building footprint dataset derived from high resolution imagery^50^, filtering out footprint features below 10 m^2^ of surface to remove potential false positives.

The shape factor of individual building features are aggregated to produce the analytics shown in the Results section, by median over 100 × 100 km global tiles for Fig. 2, and by mean in 1 × 1 km and 100 × 100 m grid cells for the other figures and analyses. The dominant construction epoch is already available at aggregated level (100 m and 1 km resolution) from GHS-AGE^51^, estimating the decade during which at least 50% of the built-up surface mapped in 2020 first becomes observable in satellite imagery, marking the dominant construction epoch of the buildings within a grid cell.

The Degree of Urbanisation settlement model, which is a global model of the rural-urban continuum at kilometric resolution^52^, was used for the year 2020 and retrieved from GHS-WUP-DEGURBA release 2025^53,54^; the global built-up surface at 1 km resolution from GHS-WUP-BUILT-S^55^ is used to subdivide the built-up stock by Degree of Urbanisation in Fig. 3. Gridded global population data for 2020 and population projections until 2100 are also issued from the Global Human Settlement Layer GHS-WUP-POP^33^, allowing for building shape factor and construction epoch figures breakdown by population, at the same resolution of 1 km. Finally, mean aggregated values for heating and cooling degree days are downloaded from the Copernicus Interactive Climate Atlas^56^ over the periods 1991–2020, 2021–2040 (near term projection), 2041–2060 (medium term projection), 2081–2100 (long term projection). ERA5 atmospheric reanalysis is used for the 1991–2020 period, and CMIP6 climate models driven by the Shared Socio‑Economic Pathways are applied for projections covering 2021–2100. Population data are extracted for a representative year of each period: the 1991‑2020 interval uses the 2020 population, the 2021‑2040 interval uses the 2030 population, the 2041‑2060 interval uses the 2050 population, and the 2081‑2100 interval uses the 2100 population.

Socio-economic indicators compared against compactness trajectories were obtained from the Urban Centre Database (UCDB R2024^57^).

### Why is compactness important?

The importance of construction epoch as an estimator for building energy efficiency is directly related to the evolution of energy efficiency policies and measures over time^21,22^. Less obvious is the contribution of compactness, through the shape factor indicator, sometimes referred to as the surface to volume ratio, defined as the ratio between the outdoor exposed building envelope surface (S) and the conditioned volume (V). A lower surface to volume ratio indicates more compact buildings: in city centers, which are typically dense and high-rise, buildings are generally characterized by lower shape factor values than those in the city outskirts or in rural areas. Notably, shape factors above 0.8–1 can pose significant challenges to achieving high energy efficiency standards, both in heating^58,59^ and in cooling regimes^60^, even with adequate thermal insulation. In contrast, higher shape factors can facilitate increased solar energy generation, through integration of solar photovoltaics in building’s envelope^61,62^. Several countries, such as Austria, Germany, Czechia, and the United Kingdom ^63,64^, have incorporated shape factor considerations into their mandatory or voluntary building standards. Various alternatives to the traditional shape factor are reported in the literature, including the Heat Loss Form Factor (HLFF)^65,66^, which utilizes floor space instead of heated volume.

In GHS-OBAT^48^, the building shape factor (Eq. 1) was calculated at feature level, approximating the ratio of the building outer surface (S) to the building volume (V) at LOD1 (simple extrusion, i.e. “shoebox”, which neglects the roof shape). With * A* footprint area, * P* footprint perimeter, both derived from the building footprint geometry, and * h* mean building height attribute, the shape factor results in:

1 $$shape\,factor = \frac{S}{V} = \frac{{2 \cdot A + P \cdot h}}{{A \cdot h}} = \frac{{2A}}{{A \cdot h}} + \frac{{P \cdot h}}{{A \cdot h}} = \frac{2}{h} + \frac{P}{A}~\quad \quad \left[ {1/m} \right]$$

**Fig. 10:**
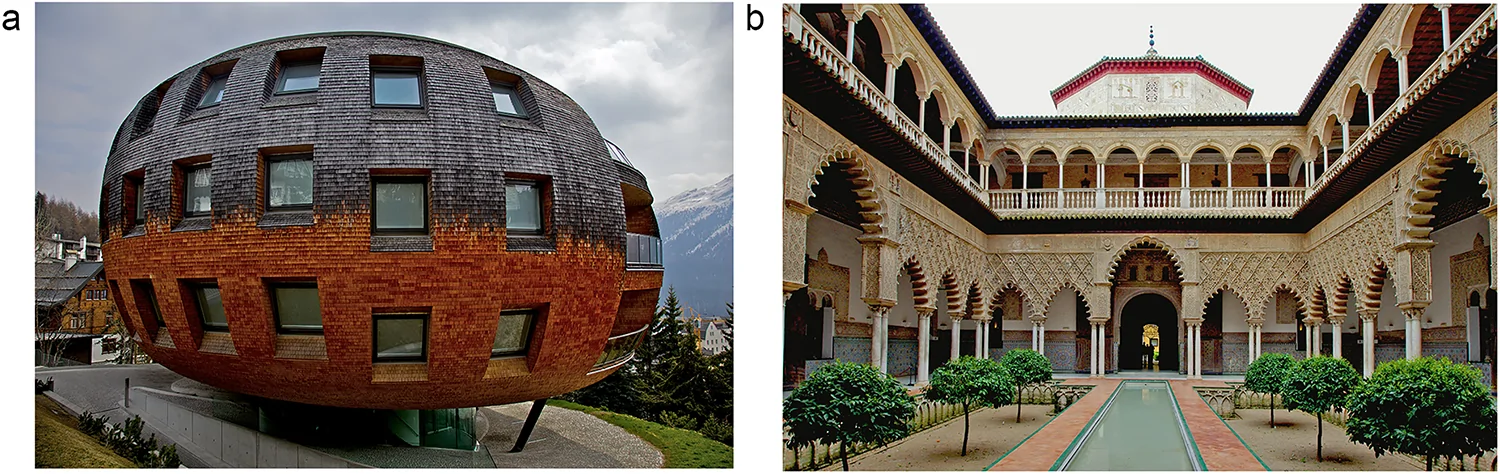
(**a**): Chesa Futura (2004), by the architect Norman Foster in St. Moritz (Switzerland), limits the outdoor exposed surface in a cold climate by approximating a sphere (photo credits: Thomas Guignard via Flickr, CC-BY-NC-SA 2.0). (**b**): the Royal Alcazar (1360) in Seville (Spain), increases outdoor exposed surface to benefit from natural ventilation, shading and evapotranspiration from vegetation in a hot climate (photo credits: Kiko Leon via Wikimedia Commons, CC-BY-SA-4.0).

In general, the energy needs to climatize a building (either in heating or in cooling regime) depend on several factors: form factors, say the conditioned volume layout and arrangement of its interface with the outdoors; thermophysical factors relative to construction materials and their assembly techniques; climatic factors describing the outside conditions, and utilization factors relative to the operation of the building. An accurate estimate of the energy needs requires analyzing such aspects into detail; on top, translating the energy needs into energy use would require data about the building energy systems (i.e. heating, ventilation, air conditioning, etc.). Gathering all this information is not possible here, given the scale of the stake and the lack of information. The shape factor addresses only one parameter, say the form of the building, describing how the conditioned volume relates with the building envelope surface. Independently from other factors, a bigger conditioned volume corresponds to higher energy needs, so larger buildings consume more energy than smaller one with the same characteristics. However, to a given volume may correspond different possible extents of envelope surface exposed to the outdoors (the limit case is the one of the spheres, with the smallest outer surface in relation to its volume, being considered the most energy efficient shape). For the same conditioned volume, a building with more exposed envelope (higher S/V shape factor), consumes more energy than a building with less exposed envelope (lower S/V shape factor) on a yearly basis, considering all the other characteristics the same. Overall, segregating more the indoor conditioned volume from the outside conditions is a good strategy, especially in cold climates (Fig. 10, left), but as the outer surface is the interface of both heat losses from and heat gains to the buildings, there are cases in which increasing such surface is more favorable. Courtyards and porches are typical examples of increased heat exchange with the outdoors via an augmented areal interface (Fig. 10, right), with the purpose of passively (i.e. without any mechanical system) cooling off the buildings in hot climates. Conversely, glazed façades, greenhouses and convective walls benefit from an increased surface exposure to solar radiation in cold climates. In both cases though, when a mechanical system intervenes, the temperature gradient between the indoors and the outdoors may increase to a point that makes passive conditioning techniques ineffective.

The bounds used to bin shape factor values in figures and maps are determined by the distribution of average kilometric shape factors over the globe (see Fig. S. 3): the very compact upper threshold is fixed at the 1 st percentile, rounded to the first decimal, which is 0.6 m^− 1^. Similarly, beyond the 99th percentile, rounded to 2.0 m^− 1^, buildings are considered very loose. The intermediate classes are obtained by selecting the middle value between 0 and 2, that is 1 m^− 1^, which coincides with the limit for an efficient climatization according to the literature (see the Introduction section). By adding or subtracting the standard deviation (0.34 m^− 1^, approximated to 0.4 m^− 1^), we conveniently retrieve 1.4 m^− 1^ as an upper bound for the medium compactness and as a lower bound for loose buildings; concurrently, we gather 0.6 m^− 1^ again, as a lower bound for compact buildings (see legend in Fig. 2 etc.).

### K-means cluster analysis of city-level age-compactness relationships

The city-level cluster analysis of the relationship between building age and compactness utilizes gridded data at 100 m resolution, comprising average building compactness from GHS-OBAT and the estimated construction epoch from GHS-AGE. Integration with the Degree of Urbanisation settlement model enables the extraction of grid cell values within urban centers (UCs) as of 2020, with cities having sparse building footprint data coverage excluded (Supplementary Table S. 3). Median shape factors are calculated per 5-year building age class and smoothed into 5-year bins from 1975 to 2020 using a 25-year moving mean filter. After normalization of the resulting time series to the range [0,1] these 10-dimensional descriptors of relative compactness trends per UC are input to the time-series k-means clustering algorithm ^67^. The Elbow method^68^ determines the optimal number of clusters, which is approximately four (Supplementary Fig. S. 13).

Sensitivity analyses using alternative city delineation methods (Supplementary Fig. S. 14) and yield consistent findings (Supplementary Fig. S. 15), although exhibiting, in part, different geographic patterns when using time series of absolute, rather than relative shape factor measures  (Supplementary, Fig. S. 16). Leveraging the t-distributed stochastic neighbor embedding method (t-SNE^69^), a 2D similarity space is produced from the 10-dimensional time-series descriptors (Fig. 5), with a perplexity parameter of 50. Socio-economic data from the Urban Centre Database (UCDB R2024), including the Human Development Index (HDI), are joined to the resulting data and averaged within 2D bins in the t-SNE similarity space (Fig. 5b-g).

### The logic of climatization vulnerability

In the context of risk management, most of the literature expresses risk with the equation: Risk = Hazard × Exposure × Vulnerability. With regards to climatization (i.e. space heating and cooling), the risk of a territory having high energy needs depends primarily on climate^70^, which may be interpreted as the hazard component. The cumulative floor space or conditioned volume of buildings in that territory, or alternatively the population occupying such buildings may translate into the exposure component. The compactness and construction epoch (age) of buildings may constitute the vulnerability descriptors, partially addressing the mentioned form and thermophysical factors driving the energy needs. Utilization and operation factors may be left out of the risk equation, being largely variable and unpredictable, i.e. depending on the specific situation of each building.

In this spirit, the climatization vulnerability is assessed at gridded level, by overlaying raster data at the resolution of 1 km, i.e. the one of the climate classification layer. The vulnerable grid cells are identified by two criteria: (1) kilometre‑scale cells that already had at least 50% of their 2020 built‑up area before 1980, and (2) cells whose shape factor exceeds a threshold linked to heating and cooling degree‑days. Cells that satisfy both conditions are classified as vulnerable.

Condition 2 relies on the estimation of the volume-specific heat transmission through the envelope $\:{Q}_{t}/V$, during the heating or cooling season. It is a rather simplified formulation that does not consider other components of the building thermal balance, i.e. ventilation, internal and solar heat fluxes, but deemed suitable for this global analysis:

2 $$\:\frac{{Q}_{t}}{V}=U\cdot\:\frac{S}{V}\cdot\:\frac{24}{1000}\cdot\:DD\hspace{1em}\hspace{1em}\left[\frac{kWh}{{m}^{3}}\right]$$

With * V* being the conditioned volume, * U* the average thermal transmittance (U-value) of the building envelope, * S* the envelope surface, 24 h in a day that retrieves the energy carried with the thermal flux, 1000 the conversion factor from Wh to kWh, and * DD* the annual heating or cooling degree days. By fixing a standard, universal floor height $\:{h}_{floor}$, the heat transmission can be expressed as floor surface * FS* -specific instead of volume-specific, which is commonly referred to as the climatization energy intensity due to heat transmission through the building thermal envelope $\:E{I}_{t}$:

3 $$\:E{I}_{t}=U\cdot\:\frac{S}{V}\cdot\:\frac{24}{1000}\cdot\:DD\cdot\:{h}_{floor}\hspace{1em}\hspace{1em}\mathrm{as}\hspace{1em}\hspace{1em}E{I}_{t}=\frac{{Q}_{t}}{FS}={Q}_{t}\cdot\:\frac{{h}_{floor}}{V}\:\hspace{1em}\hspace{1em}\left[\frac{kWh}{{m}^{2}}\right]$$

For a reference U-value $\:{U}_{ref}$, the maximum shape factor $\:{\left[S/V\right]}_{t}$, above which a given target climatization energy intensity $\:E{I}_{t}$ is exceeded for the local temperature conditions expressed in annual degree-days corresponds to:

4 $$\:{\left[\frac{S}{V}\right]}_{t}=\frac{E{I}_{t}}{{U}_{ref}\cdot\:\mathrm{max}\left(HDD,CDD\right)\cdot\:{h}_{floor}}\cdot\:41.7\hspace{1em}\hspace{1em}\left[\frac{1}{m}\right]$$

 

The target climatization energy intensity defines the maximum transmission‑related energy demand per unit of floor space, based on the reference U‑value and local temperature conditions; it is exceeded when the shape‑factor surpasses its threshold. For this work, we arrange the target climatization energy intensity at 100 kWh/m^2^ per year, for a reference U-value of 0.3 W/(m^2^ K). This choice corresponds to achieving a “B” or “C” energy label, for many countries in Europe^71^, with a very well insulated building, but can be easily compared to any other building prescription using the same unit. The reference U-value would meet most national standards in the European Union^72^ but can be related to other building codes worldwide^29^, see Supplementary Fig. S. 8. Besides, such choices stabilize the global vulnerable population in 2020 (see sensitivity analysis in Supplementary Sect. 6).

Whenever such threshold, expressed in Eq. 4, is exceeded by the average shape factor computed for the grid cell, condition 2 is met to trigger vulnerability.

The combination of both conditions identifies vulnerable kilometric grid cells, weighted by their population resulting from GHS-WUP-POP for a given epoch. The distribution of vulnerable population is shown in Fig. 8, by applying a gaussian kernel density estimation (KDE) to centroids of 100 km aggregated grid cells, weighted by their respective vulnerable population sum. A fixed bandwidth factor equal to 0.4 was selected for the gaussian kernel across all periods and scenarios.

## Conclusion

This study demonstrates that building compactness and construction epoch are effective, globally available proxy variables for assessing the energy efficiency performance of the built environment. This work provides an unprecedented,  comprehensive analysis of global building footprint data, using a shape factor metric, combined with novel construction epoch information obtained from settlement evolution patterns. By integrating open‑source, satellite‑derived datasets with climate, demographic and socio‑economic information, we quantified a novel “climatization vulnerability” indicator that highlights where older, loosely compact buildings intersect with adverse climate conditions, as of today and in future scenarios.

The spatial analysis reveals pronounced contrasts. Highly compact structures dominate dense urban centers in East Asia, the Persian Gulf and much of Europe and North America, whereas loose, low‑rise buildings are prevalent in Sub‑Saharan Africa, South‑Asia and parts of Latin America. Older building stocks (pre‑1980) are concentrated in high‑income regions, yet their vulnerability is amplified in cold climates where heating demand is high. In low‑income countries, rapid urbanization has produced a surge of recent, lightweight constructions that are often loosely shaped, leading to heightened cooling demand under future warming scenarios.

Cluster analysis of more than 9,000 urban centers identified four distinct compactness‑age trajectories, strongly linked to regional income levels and urbanization patterns. These trajectories provide a basis for targeted policy measures. In high‑income, regulated markets, retrofitting envelopes (e.g., window upgrades, insulation) can achieve substantial heating‑energy savings. In rapidly urbanizing low‑income regions, setting minimum compactness standards, promoting high thermal‑mass construction techniques and encouraging passive ventilation design are essential to curb the projected rise in cooling demand.

The spatial distributions of vulnerability suggest that, under most optimistic socioeconomic pathways (SSP 1–1.9, SSP 1–2.6, SSP 2–4.5), the global population exposed to old and ill-shaped building stock could grow by 10%−30% by 2100, from circa 50 million today, substantially shifting from cold regions of Eurasia to the Sahel zone, Gulf countries and Pakistan. This underscores the urgency of integrating building‑form considerations into climate‑adaptation and energy efficiency strategies.

Limitations of the analysis include the reliance on simplified shape‑factor calculations affected by the accuracy and comprehensiveness of building footprint mapping and height estimation; uncertainty about renovation rates; exclusion of climatization system efficiency, cultural adaptation and occupant behavior, that all have a considerable impact on energy demand for climatization. Nevertheless, the approach offers a reproducible framework for large‑scale monitoring and prioritization of renovation programs, that could be integrated with finer-scale Urban Building Energy Modelling.

Future research should refine compactness metrics, incorporate dynamic occupancy and system‑efficiency data, and evaluate the cost‑effectiveness of climate‑responsive design interventions at national and sub‑national levels. By doing so, policymakers can better align building‑stock transformation with global climate‑neutrality targets.

## Authors' note

The designations employed and the presentation of materials and maps do not imply the expression of any opinion whatsoever on the part of the European Union concerning the legal status of any country, territory or area or of its authorities, or concerning the delimitation of its frontiers or boundaries that if shown on the maps are only indicative. The boundaries and names shown on maps do not imply official endorsement or acceptance by the European Union. The views expressed herein are those of the authors and do not necessarily reflect the views of the European Union. Authors declare no conflict of interest.

## Supplementary Information

Below is the link to the electronic supplementary material.

Supplementary Material 1

## Data Availability

Feature-level building data is available in the GHS-OBAT R2024A dataset (CSV or GPKG format)^25^, integrated with East Asian building footprint geometries^26^. Global built-up surface data is available from GHS-WUP-BUILT-S R2025A^55.^ Population estimates for 2020 and projections until 2100 belong to GHS-WUP-POP R2025A^33^. Global Degree of Urbanisation at grid level is available from GHS-WUP-DEGURBA R2025A^54^, and global grids of dominant building construction epoch are gathered in GHS-AGE R2025A^73^. Socio-economic indicators and urban center geometries are issued from the UCDB R2024^57^. Global average shape factor values for compactness are available at raster level, at the resolution of 1 km and 100 m from the following public link, which is part of the GHS-OBAT R2024A dataset^25^: https://jeodpp.jrc.ec.europa.eu/ftp/jrc-opendata/GHSL/GHS_OBAT_GLOBE_R2024A/GHS_OBAT_GRIDSTATS_GLOBE_R2024A/V1-0/.
