# Supplementary material for: A global, proxy-based assessment of building climatization vulnerability
Source: Sci Rep. 2026 Jul 28;16:22656. doi: 10.1038/s41598-026-58888-y (PMC13416028; doi:10.1038/s41598-026-58888-y)
Supplement: Supplementary file 1 — Supplementary Material 1 [file 41598_2026_58888_MOESM1_ESM.docx]

Supplementary material

A global, proxy-based assessment of building climatization vulnerability

[Florio, P.](https://orcid.org/0000-0001-7866-7401) ^1^*, [Uhl, J.H.](https://orcid.org/0000-0002-4861-5915)^1^, [Politis, P.](https://orcid.org/0000-0001-6417-1587)^3^, [Melchiorri, M.](https://orcid.org/0000-0002-3009-8868)^1^, [Maduta, C.](https://orcid.org/0000-0002-0922-3740)^1^, [Krasnodębska, K.](https://orcid.org/0000-0002-1398-9095)^2^, [Martinez, A.M.](https://orcid.org/0000-0002-4220-8358)^1^

^1^ European Commission, Joint Research Centre (JRC), Ispra, Italy
^2^ Stanisław Leszczycki Institute of Geography and Spatial Organization, Polish Academy of Sciences, Warsaw, Poland

^3^ European Dynamics Belgium S.A., Brussels, Belgium

* corresponding author, email address: [pietro.florio@ec.europa.eu](mailto:pietro.florio@ec.europa.eu)

1. Completeness assessment of building footprints

The building footprint database in vector format used to produce global maps of compactness (shape factor) is the mentioned GHS-OBAT R2024A [25], which relies on footprint geometry from Overture buildings release 2024-07-22.0^[[1]](#footnote-2)^. As mentioned in the data descriptor accompanying GHS-OBAT [48], the coverage of such release of Overture buildings is not complete, especially in Eastern Asia, when compared against GHS-WUP-BUILT-S R2025A [55] as reference data. To overcome this limitation, an open building footprint dataset derived from very high resolution imagery in East Asia ￼[26]are integrated in the building footprint database, filtering out small footprint features under 10 m^2^: this paves the way to a new version of GHS-OBAT, using a more up-to-date release of Overture buildings which already integrates such data.


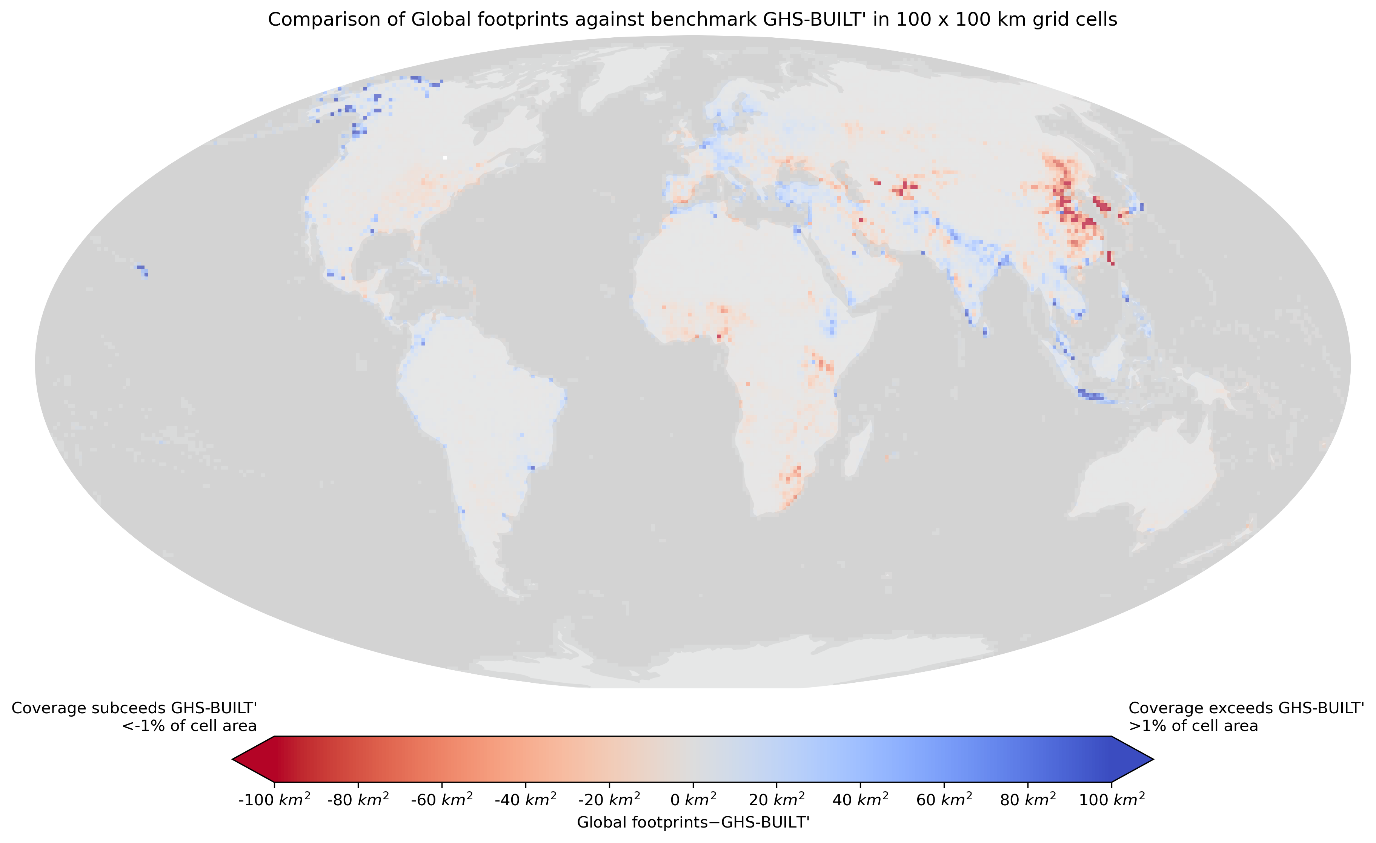


Figure S. 1. GHS-OBAT (Overture derived + East Asia patch) building footprint surface benchmarking against adjusted GHS-BUILT-S, a product issued from the analysis of satellite imagery. Colors represent the difference in surface km^2^ between the aggregated footprint surface and adjusted GHS-BUILT-S in 100 × 100 km tiles. Maps created with Cartopy, with free vector and raster basemap data from Natural Earth @ naturalearthdata.com.

1. Population breakdown by Degree of Urbanisation in geographic regions

The bar charts in Figure S. 2 provide synthetic detail to the maps in the Results section. It emerges with clear evidence how differences between urban and rural areas are more striking in certain regions. For example, in Latin America and the Caribbean (LAC) as well as in Eastern and South-Eastern Asia (ESEA), compact buildings (shape factor < 1) host circa 80% of the population in urban areas, but less than 10% in rural areas. In other regions, like Oceania and Sub-Saharan Africa, the share of urban population living in compact buildings is close to 50% only. In terms of construction epoch, the extremes are Europe with 65% rural population vs 80% urban population living in old buildings (built before 1980) and Sub-Saharan Africa, with slightly more than 10% rural population vs circa 30% urban population. The most remarkable disparity between urban and rural areas happens in Australia and New Zealand, with 70% urban population vs 30% rural population living in old buildings.

a

LAC

Latina America and the Caribbean
CSA

Central and Southern Asia
SSA

Sub-Saharan Africa
E

Europe
NAWA

Northern Africa and Western Asia
O

Oceania
ANC

Antarctica and Caspian sea
ANZ

Australia and New Zealand
NA

Northern America
ESEA

Eastern and South-Eastern Asia
#A

Asian disputed territories


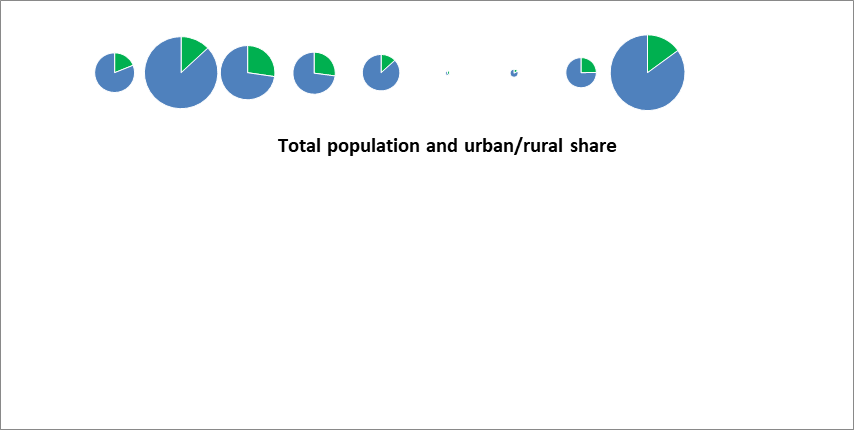


urban

rural

b

Figure S. 2. Share of population in 2025, by UN SDG Region, main degree of urbanisation (GHS-WUP-DEGURBA 2020) and (a) bins of shape factor, (b) construction epochs

1. Shape factor distribution

The average shape factor in 1 km resolution grid cells over the world has been used, along with energy efficiency considerations, to set the bounds for compactness categories. The distribution is shown in Figure S. 3.


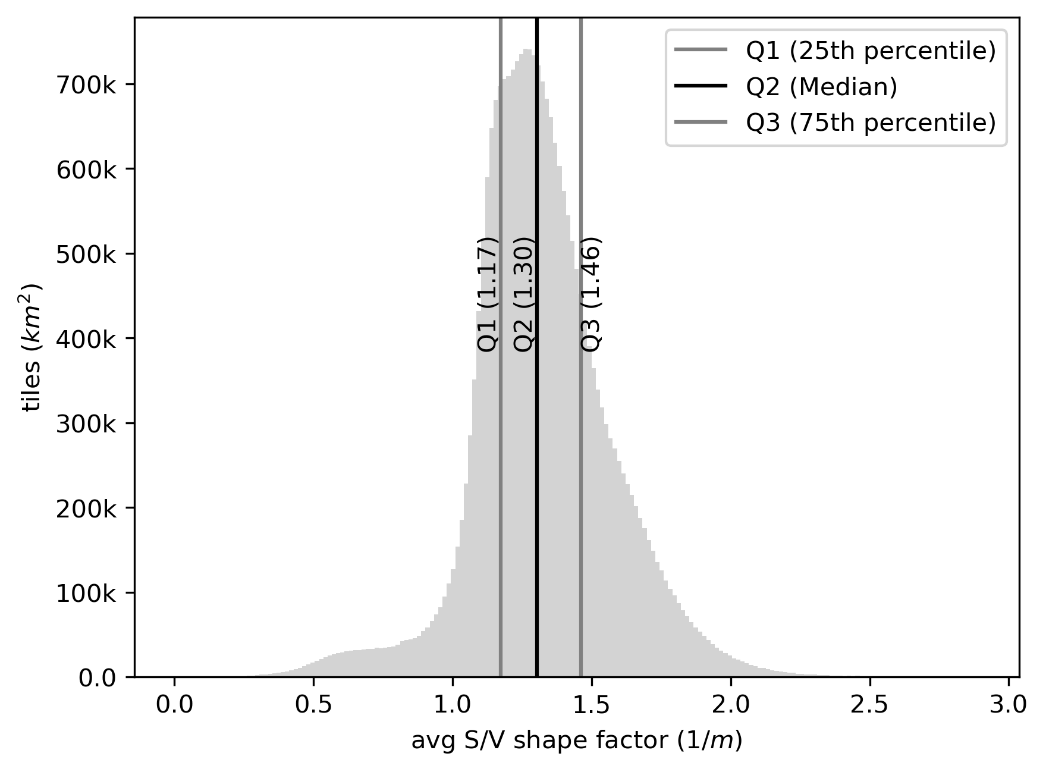


Figure S. 3. Distribution of the average shape factor in 1 km resolution grid cells over the world.


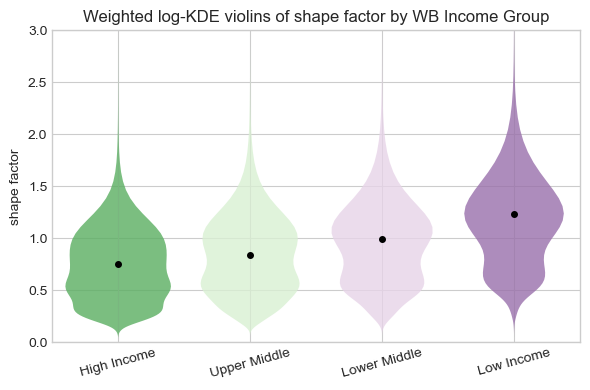


Figure S. 4. Violin plots of area-weighted building shape factor by World Bank income class, using a logarithmic gaussian kernel. The black dot represents the median.

In low-income countries (LICs), buildings are less compact on average (see Figure 6 and Figure 7). However, there is also a notable presence of compact buildings (with a shape factor of 0.6-1, see Figure S. 4): this creates a secondary peak in the shape factor distribution, unevenly skewed around the shape factor extremes, rather than in medium shape factor ranges, in contrast to higher-income countries.

1. Epoch distribution

With regards to building age, findings in Figure S. 5 indicate that middle urbanization categories in 2020 host more recent buildings in proportion to other categories, with a minimum of circa 20% pre-1980 built-up surface in suburban and peri-urban areas. This share increases bidirectionally up to more than 50% in urban centers and almost 60% in very low-density rural areas. Conversely, the presence of more recent constructions (after 2000) is lightly skewed towards rural areas, but with remarkable shares in suburban and peri-urban areas (more than 30%), as transformative territories with increasing densification. Most recent buildings (after 2010) lie mainly in low density rural grid cells (Figure S. 5). It is not surprising, in a real estate investment optic, that suburban areas are preferred for new constructions to urban in-fills, even if the latter may be under-represented in our data as more difficult to detect and isolate from the urban core.

Figure S. 5. Share of global built-up surface by gridded Degree of Urbanization level 2 (GHS-WUP-DEGURBA) for 2020 and by construction epoch. Bars are labelled with surface values in m^2^.

The combination of compactness and age observations shows that more recent buildings are less compact, especially in urban areas (Figure S. 6), where 70% of built-up surface constructed after 2010 is medium to loose (>1): this share is lower in older epochs (15% before 1980, 50% in 1980s, 25% in 1990s and 40% in 2000s). This is another consequence of urban sprawl, as recent suburban developments seem to be composed of uncompact buildings. Another important finding lies in the disparity between rural and urban areas, as in rural areas this trend is much less evident. Table S. 1 shows into detail that most people globally live in buildings that are old (< 1980) and in a compact range (0.6-0.8), especially in urban areas, or old and medium to loose (>1), which host almost equally spread population between rural and urban areas. In these cases, to facilitate interpretation, the very low and low density rural grid cells and the rural clusters have been grouped together as “rural”, and the other classes as “urban”.

Figure S. 6. Share of global built-up surface in 2020 by bins of shape factor, main degree of urbanization (GHS-WUP-DEGURBA 2020) and construction epochs

Table S. 1. Share of global population in 2020 by bins of shape factor, main degree of urbanization (GHS-WUP-DEGURBA 2020) and construction epochs

| construction epoch | | <1980 | 1980-1990 | 1990-2000 | 2000-2010 | 2010-2020 | **TOT** |
| --- | --- | --- | --- | --- | --- | --- | --- |
| S/V compactness factor bins | | Share of global population | | | | |  |
| 0-0.2 |  | **0.44%** | **0.03%** | **0.10%** | **0.06%** | **0.02%** | **0.65%** |
|  | rur | 0% | 1% | 0% | 1% | 2% | 1% |
|  | urb | 100% | 99% | 100% | 99% | 98% | 99% |
|  |  |  |  |  |  |  |  |
| 0.2-0.4 |  | **5.29%** | **0.40%** | **0.94%** | **0.61%** | **0.18%** | **7.41%** |
|  | rur | 1% | 1% | 1% | 1% | 2% | 1% |
|  | urb | 99% | 99% | 99% | 99% | 98% | 99% |
|  |  |  |  |  |  |  |  |
| 0.4-0.6 |  | **11.34%** | **1.18%** | **2.24%** | **1.41%** | **0.34%** | **16.52%** |
|  | rur | 1% | 2% | 1% | 2% | 3% | 1% |
|  | urb | 99% | 98% | 99% | 98% | 97% | 99% |
|  |  |  |  |  |  |  |  |
| 0.6-0.8 |  | **14.32%** | **2.41%** | **3.81%** | **2.48%** | **0.56%** | **23.59%** |
|  | rur | 3% | 4% | 3% | 2% | 3% | 3% |
|  | urb | 97% | 96% | 97% | 98% | 97% | 97% |
|  |  |  |  |  |  |  |  |
| 0.8-1 |  | **6.25%** | **1.93%** | **2.11%** | **1.67%** | **0.68%** | **12.68%** |
|  | rur | 13% | 11% | 10% | 9% | 12% | 11% |
|  | urb | 87% | 89% | 90% | 91% | 88% | 89% |
|  |  |  |  |  |  |  |  |
| >1 |  | **13.20%** | **7.34%** | **4.20%** | **5.95%** | **7.85%** | **39.15%** |
|  | rur | 53% | 29% | 32% | 33% | 37% | 39% |
|  | urb | 47% | 71% | 68% | 67% | 63% | 61% |
|  |  |  |  |  |  |  |  |
| **TOT** |  | **50.84%** | **13.28%** | **13.40%** | **12.19%** | **9.63%** | **100.00%** |
|  | rur | 17% | 19% | 13% | 18% | 31% | 18% |
|  | urb | 83% | 81% | 87% | 82% | 69% | 82% |
|  |  |  |  |  |  |  |  |

1. Climate

Outdoor climate has certainly an impact on construction materials and techniques, and this is reflected in the analysis of building compactness and age by climate class (Figure S. 7). Least compact buildings find predominantly place in tropical climate, where both temperature and humidity are high all year long. The need for frequent windows opening, open spaces and cross-ventilation, makes large, spreading buildings adapted for an adequate climate control strategy. Most of the buildings in dry, hot climate (Bwh and Bsh) tend to lower shape factors, to collect water more efficiently, confine and disperse heat. In dry cold climate conditions (Bwk and Bsk), buildings tend to be more compact, at least in urban areas (the majority in the 0.4-0.6 shape factor range): this confirms outdoor temperature as a major driver for compactness.

Compact buildings belong mostly to temperate and cold classes, with some different distributions though. In the temperate region, the urban – rural divide in compactness strikes more strongly: in urban territories, most buildings are compact (shape factor <1), differently from rural areas, where the large majority is less compact (shape factor >1). However, temperate zones with a hot summer (Cfa) account for larger shares of compact buildings compared to temperate zones with a less hot, warm summer (Cfb). Overall, still, temperate regions host an increasing proportion of buildings along with growing compactness (and lower shape factors). Cold climate with dry winter (especially DWa) dominates among very compact buildings (shape factor < 0.6), while cold climate without dry season (Dfa and Dfb) peaks in the compact range (0.6-1.0).

It is more difficult to identify a clear gradient in the share of buildings by climate zones across construction epochs. Newer buildings tend to lie more in tropical climates (A), whereas older buildings in colder classes (D).

The global raster holding the Köppen – Geiger climate classification at 0.01° resolution^[[2]](#footnote-3)^ (circa 1 km at the equator) for the epoch 1991 - 2020 was re-projected to World Mollweide, the native projection of all other aforementioned products, before being matched with them.

a

b

Figure S. 7. Share of global built-up surface in 2020, by bins of shape factor (a) or construction epoch (b), main degree of urbanisation (GHS-WUP-DEGURBA 2020) and Köppen – Geiger climate class

1. Sensitivity of climatization vulnerability

Climatization vulnerability is sensitive to assumptions about old buildings and extreme compactness levels. From Equation 4, the maximum shape factor threshold as a function of the local temperature conditions expressed in annual degree-days depends on a reference U-value $U_{ref}$, and a target climatization energy intensity $EI_{t}$. The target climatization energy intensity defines the maximum transmission‑related energy demand per unit of floor space, based on the reference U‑value and local temperature conditions; it is exceeded when the shape‑factor surpasses its threshold.

Figure S. 8a shows that the selected values for the reference U-value (0.3 W m^-2^ K^-1^) and the target climatization energy intensity (100 kWh/m^2^ per year) are rather conservative, limiting the global vulnerable population to 48 million in 2020 (baseline). Varying each parameter individually shows that raising the target climatization energy intensity (while keeping the reference U‑value at 0.3 W m^-2^ K^-1^) relaxes the energy‑demand constraint, lifts the allowable shape‑factor threshold and consequently reduces the global vulnerable population in 2020. However, such decrease is minimal with a target increase above 100 kWh/m^2^ per year. Conversely, altering the target to 50 kWh/m^2^ per year would imply a global vulnerable population growth by a factor 10 (circa 500 million).

Increasing the reference U-value for a given target climatization energy intensity translates into requiring the same energy demand, with a lower insulation level. This implies lowering the shape factor threshold to more compact, stricter values, and increasing the global vulnerable population in turn. It is particularly interesting to compare the reference U-value to legal limits in selected cities [29], shown in the swarm plot in Figure S. 8a. The baseline reference U-value of 0.3 W m^-2^ K^-1^ corresponds to the limit in Berlin, Germany. Setting it to lower values (e.g. 0.22 W m^-2^ K^-1^ for Ljubljana, Slovenia, while keeping the target climatization energy intensity at 100 kWh/m^2^ per year) would impact global vulnerable population minimally. However, a reference U-value of 0.6 W m^-2^ K^-1^ (like in Kyiv, Ukraine and Kuala Lumpur, Malaysia), would increase the global vulnerable population tenfold.

Reference U-value and target climatization energy intensity are studied for independent variation. However, they are interlinked, as they respectively appear at the denominator and the numerator of the shape factor threshold formulation in Equation 4. The baseline parameters setting marks as vulnerable those grid cells where most of the built-up surface was constructed in 1980 already, and the average shape factor implies an energy demand above 100 kWh/m^2^ per year with an insulation level close to the building code limit for Berlin, Germany (0.3 W m^-2^ K^-1^). This shape factor cutoff is equivalent to 200 kWh/m^2^ per year for an insulation close to the limit of Kyiv, Ukraine and Kuala Lumpur, Malaysia (0.6 W m^-2^ K^-1^).

Sensitivity of vulnerable population to the old construction epoch, before which the built-up surface is considered vulnerable, is more linear. Figure S. 8b highlights a 20 million people increase in vulnerable population per every decadal progress in the epoch threshold.


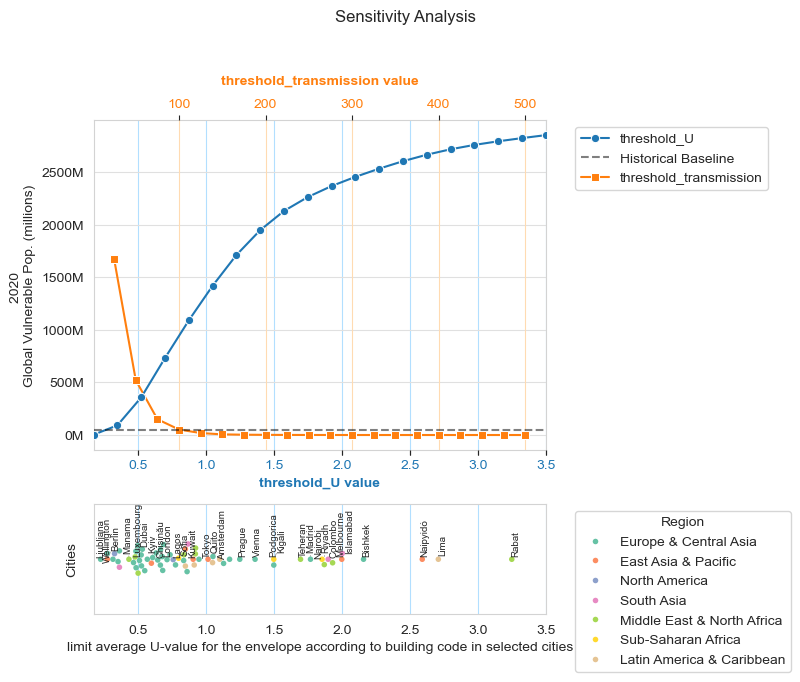

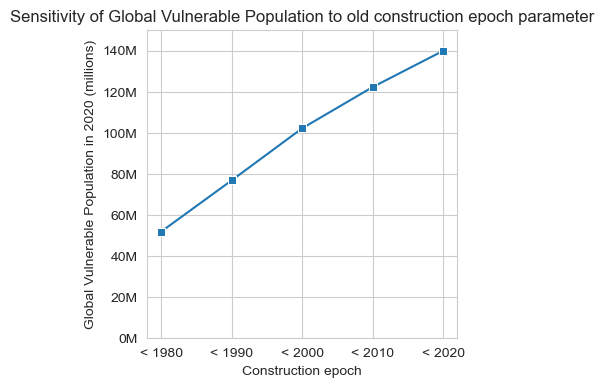


b

a

Figure S. 8. One-at-a-time (OAT) sensitivity analysis of the global vulnerable population in 2020 to parameter thresholds. (a): compactness parameters (shape factor) used in the vulnerability definition; (b): the old construction epoch parameter, before which the built-up surface is considered vulnerable. Each parameter is varied independently to understand its impact on the total vulnerable population, while the others remain steady to their default value. The horizontal dashed line intersects all parameter curves to their default value, representing the global vulnerable population in the default scenario, which stands at circa 50 million.

1. Impact of building renovation on construction epoch

Since the early 2000s, building codes have driven an intense retrofitting activity. In Europe the first Energy Performance of Buildings Directive (EPBD) was adopted in 2002, prompting Member States to impose cost‑optimal minimum‑energy standards and Energy Performance Certificates, then integrated further with the introduction of the Nearly‑Zero‑Energy Building target. In the United States, state and local adoption of the ASHRAE 90.1 energy standard (integrated via the International Energy Conservation Code) has become widespread since the mid‑2010s, making compliance mandatory for major renovations and driving a large‑scale retrofit market. Together, these regions account for the bulk of recent worldwide building‑renovation activity, considering that renovation targets appear in building energy codes from only 16 countries worldwide^[[3]](#footnote-4)^.

However, the pace at which renovations take place remains minor. The International Energy Agency estimates annual retrofitting rates at around 1%, with most of these being shallow renovations^[[4]](#footnote-5)^. The European Commission assesses a deep renovation annual rate at 0.2% on average among Member States^[[5]](#footnote-6)^, although the target is to increase it to 3% by 2030. If a 0.2 % constant deep‑renovation rate were applied to pre‑1980 buildings in both the European Union and the United States from 1980 to 2020, about 8 % of such buildings would have had their energy efficiency upgraded (4% if assuming deep renovations took place only after building renovation codes in early 2000).

The impact of deep renovation on the 2020 vulnerable population in the European Union (the former 28 Member States) and the United States can be assessed by allocating the 2020 GHS‑WUP‑POP figures for vulnerable grid cells to buildings in the GHS‑OBAT database in proportion to their volume. A random removal of 8 % of pre‑1980 buildings at national level, and the population associated with them, is then applied to represent the estimated retrofit rate, resulting in a reduction of vulnerable residents of between 3 % and 12 %.

Table S. 2. Vulnerable population in 2020, in European Union countries (EU-28) and the United States. In the middle column, 8% of buildings are assumed to be renovated and the corresponding population is subtracted. The percentage difference is in the third column.

| Country | Vulnerable population, 2020, without renovation | Vulnerable population, 2020, with renovation (8% of buildings) | Percentage Difference |
| --- | --- | --- | --- |
| AUT | 378,157 | 354,974 | -6.1% |
| BEL | 631 | 596 | -5.6% |
| BGR | 1,499 | 1,414 | -5.7% |
| CYP | - | - | 0.0% |
| CZE | 15,179 | 14,278 | -5.9% |
| DEU | 14,166 | 13,401 | -5.4% |
| DNK | 328 | 309 | -5.9% |
| ESP | 7,103 | 6,332 | -10.9% |
| EST | 191,406 | 177,874 | -7.1% |
| FIN | 1,260,145 | 1,180,189 | -6.3% |
| FRA | 129,871 | 122,154 | -5.9% |
| GBR | 1,459 | 1,376 | -5.7% |
| GRC | - | - | 0.0% |
| HRV | 51 | 51 | 0.0% |
| HUN | 75 | 67 | -10.1% |
| IRL | 25 | 25 | 0.0% |
| ITA | 187,902 | 176,786 | -5.9% |
| LTU | 62,072 | 57,805 | -6.9% |
| LUX | - | - | 0.0% |
| LVA | 202,995 | 188,751 | -7.0% |
| MLT | - | - | 0.0% |
| NLD | 6,953 | 6,739 | -3.1% |
| POL | 27,706 | 26,132 | -5.7% |
| PRT | - | - | 0.0% |
| ROU | 178,459 | 168,116 | -5.8% |
| SVK | 87,819 | 82,871 | -5.6% |
| SVN | 140 | 122 | -12.7% |
| SWE | 659,265 | 615,462 | -6.6% |
| USA | 1,493,623 | 1,390,560 | -6.9% |

1. Impact of roof shape on shape factor

Equation 1 shows the computation of shape factor for a shoebox type building volume (Level of Detail – LOD1), which assumes a flat roof. This assumption relies on the fact that attic space is uninhabited and unconditioned in many cases. In a more general situation, the shape factor can be formulated as follows:

$$shape factor=\frac{S}{V}=\frac{A_{f}+A_{r}+P\cdot\bar{h}}{A_{f}\cdot\hat{h}}=\frac{A_{f}(1+F)+P\cdot\bar{h}}{A_{f}\cdot\hat{h}}=\frac{1+F}{\hat{h}}+\frac{P}{A_{f}}\cdot\frac{\bar{h}}{\hat{h}}$$

With $A_{f}$ area of the footprint (floor), $A_{r}$ area of the roof, $\bar{h}$ eaves mean height that retrieves the façade surface, $\hat{h}$ roof mean height that retrieves the correct building volume. The factor $F$ is specific to the roof shape that distinguishes it from the standard flat roof, like the height ratio $\bar{h}/\hat{h}$, which is a fraction close to 1. In a simple gabled roof covering a rectangular footprint with two equal slopes, and ridge parallel to the longer side, $F$ is equal to the secant of the pitch angle $\sec\theta$, and the height ratio is equal to 1. In a simple hip roof with four equal slopes, ridge centered, $F=k\cdot\sec\theta$, where $k$ is a modest correction (typically 1.05-1.10) that accounts for the shorter ridge length compared with a gable roof. A Mansard roof will have two slopes and $F$ is therefore the sum of the secant of pitch angles.

To give a numerical example, we may assume a gabled roof over a simple rectangular footprint, with a roof pitch of 30°. The difference in shape factor compared to a flat roof will be:

$$\Delta\left( \frac{S}{V} \right)=\frac{F-1}{h}$$

Which in this case equals to 0.16 / $h$. Assuming a height of 3 meters, which is the lowest for a habitable building, the formula returns a difference of 0.06 1/m in shape factor compared to the flat roof, which is almost negligible. Overall, for the purposes of this work, the error in height estimates derived from remote sensing are larger than the error engendered by a roof type approximation to a flat roof, therefore not further addressed.

1. City examples

Building compactness and construction epoch driving vulnerability for example cities are shown in Figure S. 9. The spatial distribution of shape factor, ranging from very compact shape factor values in city centers to very loose values in the outskirts, is similar in many cities globally.


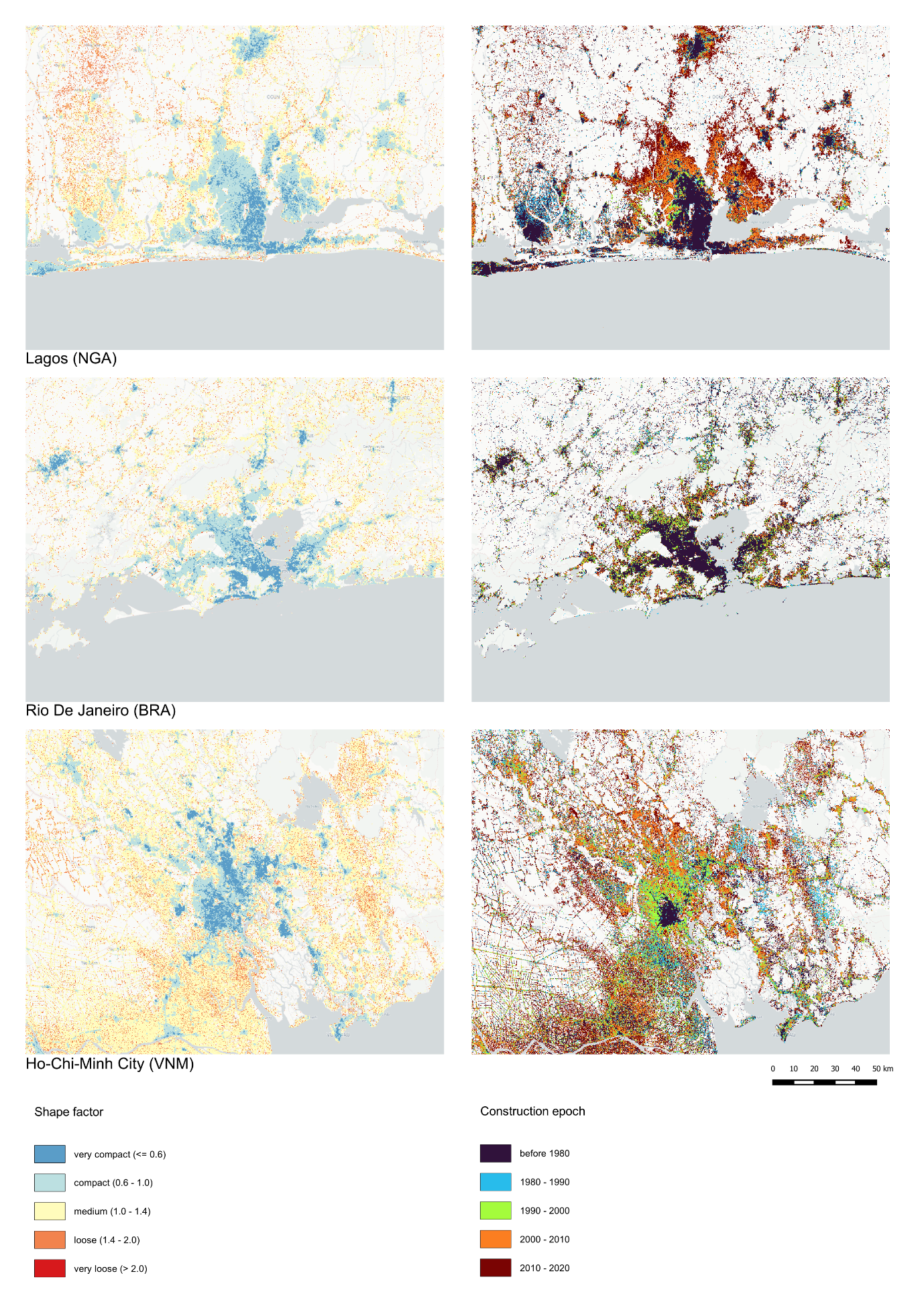


Figure S. 9. Shape factor bins (left) and construction epoch (right) for three example city areas, with highly compact and dense building clusters in main settlements, looser in the outskirts. Maps created with QGIS, with free vector and raster basemap data from Carto, under CC BY 3.0 and OpenStreetMap.

1. Cluster analysis - extended results

This section contains additional results related to the cluster analysis described in the main paper. Time-series k-means cluster analysis separates urban centers (UCs) globally in approximately four clusters.

Figure S. 10 shows cluster membership of UCs in each country involved in the analysis, and the population residing in UCs of each cluster. For each cluster, the UC with the highest Silhouette Score has been identified, representing a “typical” UC for each cluster. The underlying source data (i.e., gridded average shape factor and median building age), and examples of the urban fabric in these cities are shown in Figure S. 11. As discussed in the main text, the identified clusters show strong patterns across world regions and income classes, which is further illustrated in Figure S. 12.

These clusters were identified using the Elbow method, that identifies the number of clusters k for which the within-cluster sum-of-squares starts to drop linearly with increasing k (Figure S. 13). This “optimal” k = 4 is also found when using different spatial delineations of cities, and when using absolute, rather than normalized time series of building shape factors, as can be seen in Figure S. 13.

An example of these different spatial delineations of cities, or “urban areas”, is shown in Figure S. 14. As discussed earlier, the input data suffers from lower levels of coverage, for example in China. For this reason, UCs and other spatial entities were filtered by their completeness, and only urban areas of high to very-high data coverage were assessed (Table S. 3).

Despite the different spatial delineations of urban areas (Figure S. 14), cluster analyses yield highly similar results (Figure S. 15), with largely consistent geographic patterns.

Until here, cluster analyses are based on normalized time series of building shape factors, i.e., scaled to the range of [0,1] based on the lowest and highest shape factors per urban area, in order to capture local variations of building compactness independent of overarching, regional and income-related trends of building compactness, as reported for example in Figure 6 in the main text. For comparison, the cluster analysis based on absolute shape factor time series is consistent with these general findings, with clusters largely separating between the Global North and the Global South (Figure S. 16).


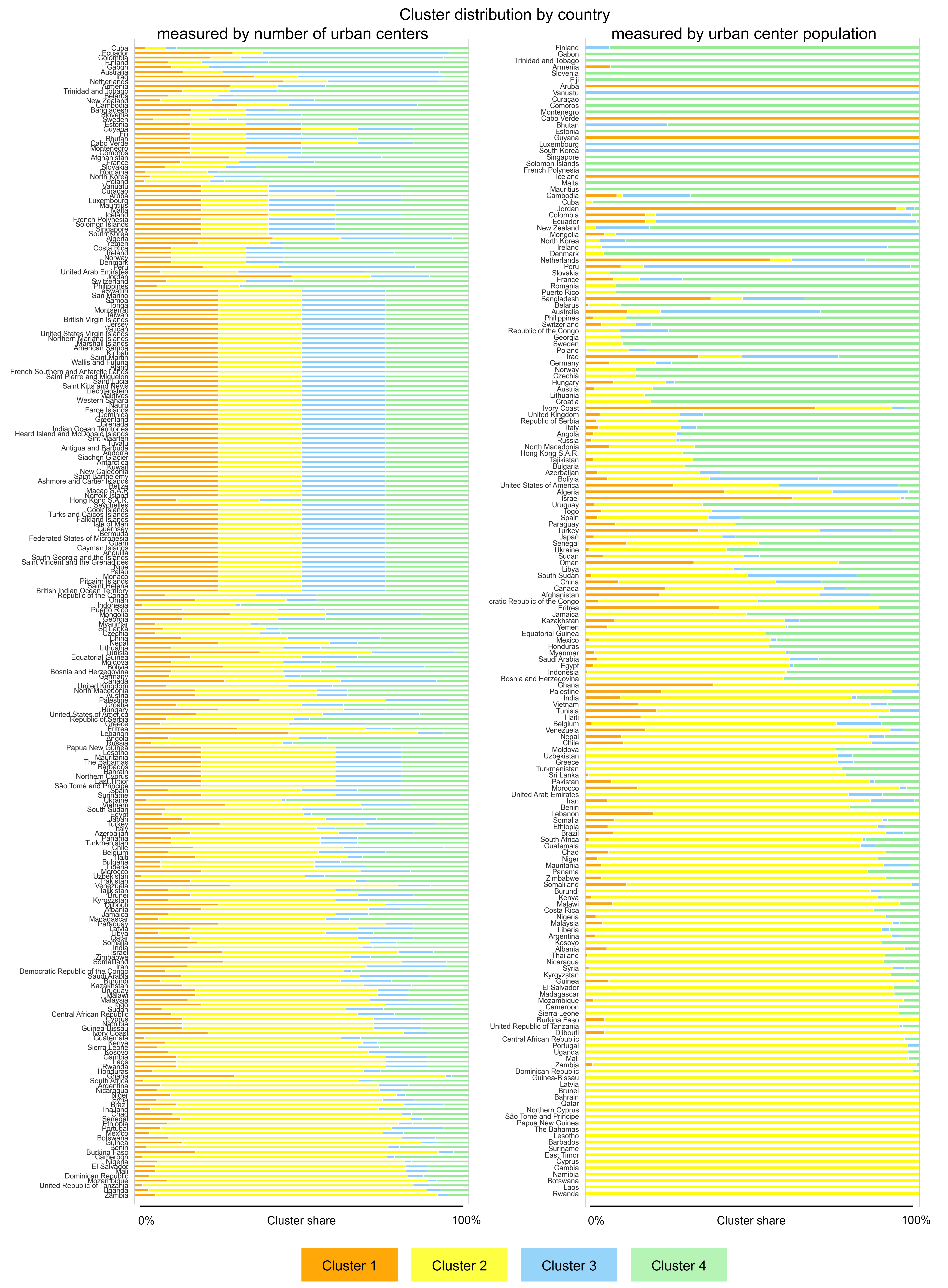


Figure S. 10. Results of time series cluster analysis of relative building compactness by age class per urban center. Cluster distribution by country, measured by the number of urban centers per cluster (left) and by their population shares (right). Vertical sorting by the share of cluster 1.


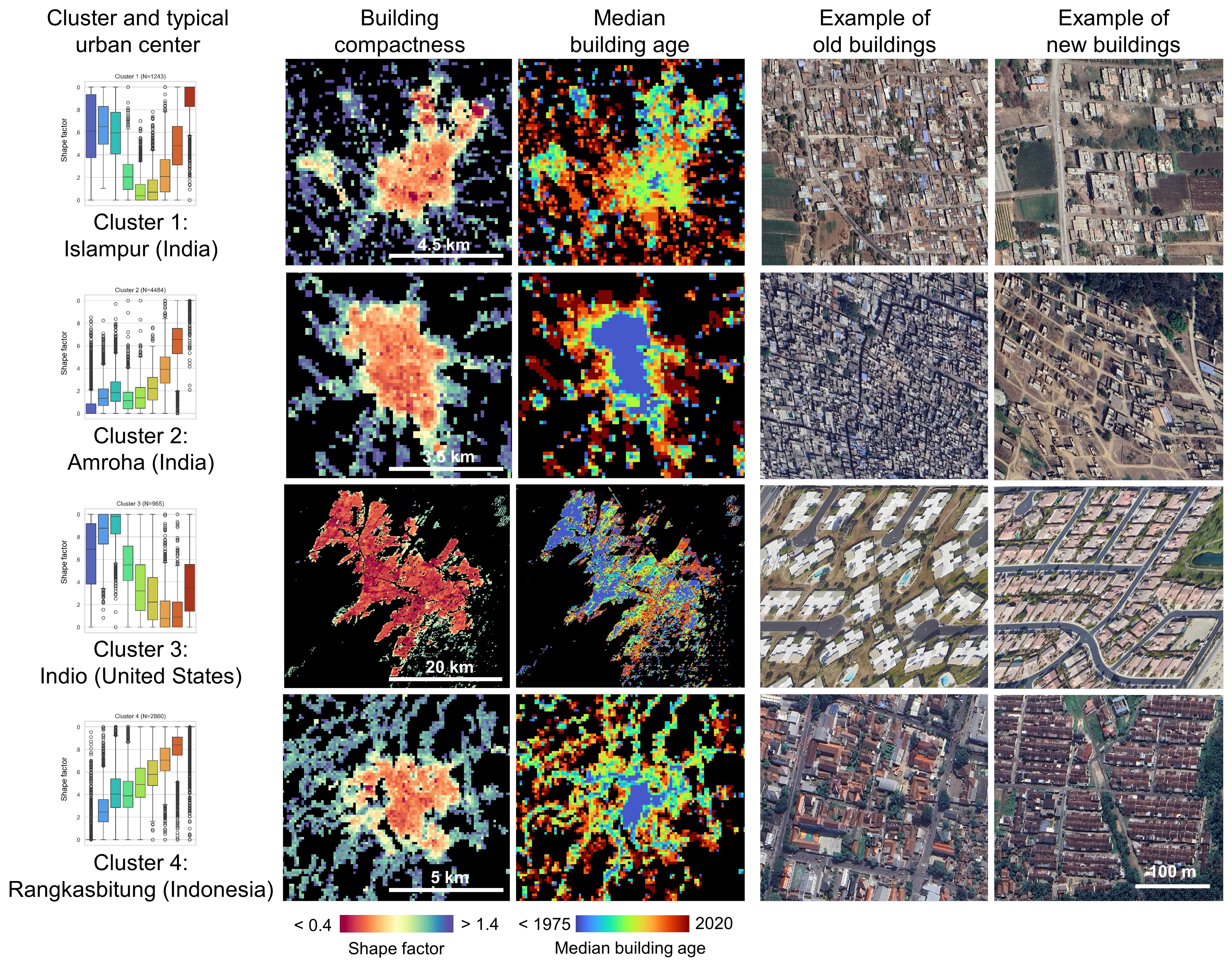


Figure S. 11. Typical urban centers for the four building age – compactness relationship types. Shown are the urban centers with the highest Silhouette score per cluster, including the respective cluster trajectory (left), the gridded, 100-m average building compactness data derived from GHS-OBAT, and gridded median building age data at 100 m resolution from GHS-AGE. Based on these gridded data, building compactness time series per urban center were extracted as input for k-means cluster analysis Also shown are examples of old (i.e., <1975) and recent (i.e., >2010) building stock, manually, randomly selected within each of the four urban centers. Maps created with Matplotlib. Source of aerial imagery: Google ©.


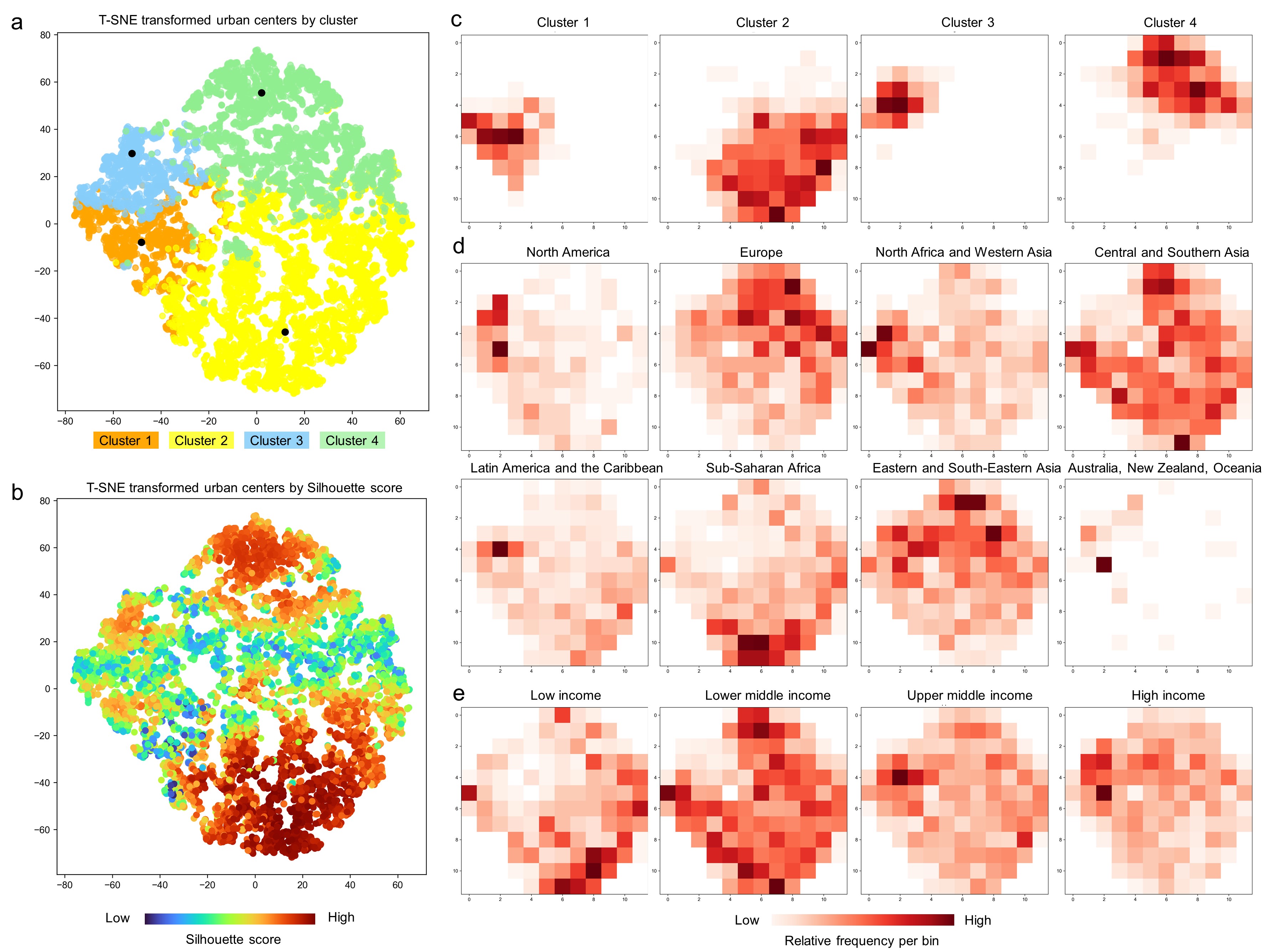


Figure S. 12. Visualizing the results of the urban center cluster analysis using t-SNE. Using time series of relative building compactness per urban center from 1975 to 2020, four clusters were identified using time-series k-means clustering. The resulting clusters are shown in a t-SNE based 2-D similarity space where urban centers with similar building compactness trends are located nearby. The four clusters are clearly grouped in the t-SNE similarity space, as shown in panel (a). Black dots in (a) represent the location of the most “typical” urban center per cluster, which are also shown in the main article Figure 4, and in the Supplementary Figure S.16. These “typical” urban centers have the highest Silhouette scores per cluster. The Silhouette score measures how similar a cluster member is to other cluster members, and how dissimilar to members of other clusters. The Silhouette scores for all urban centers are shown in (b), illustrating that low Silhouette scores are located near the edges of clusters, possibly in areas of overlap between clusters. Panel (c) shows the relative frequencies of urban centers per cluster within coarse 2-D bins imposed over the t-SNE similarity space, (d) shows relative frequencies of urban centers per world region, indicating a relatively strong association of specific building compactness trajectories with world regions, as well as with (e) income classes.


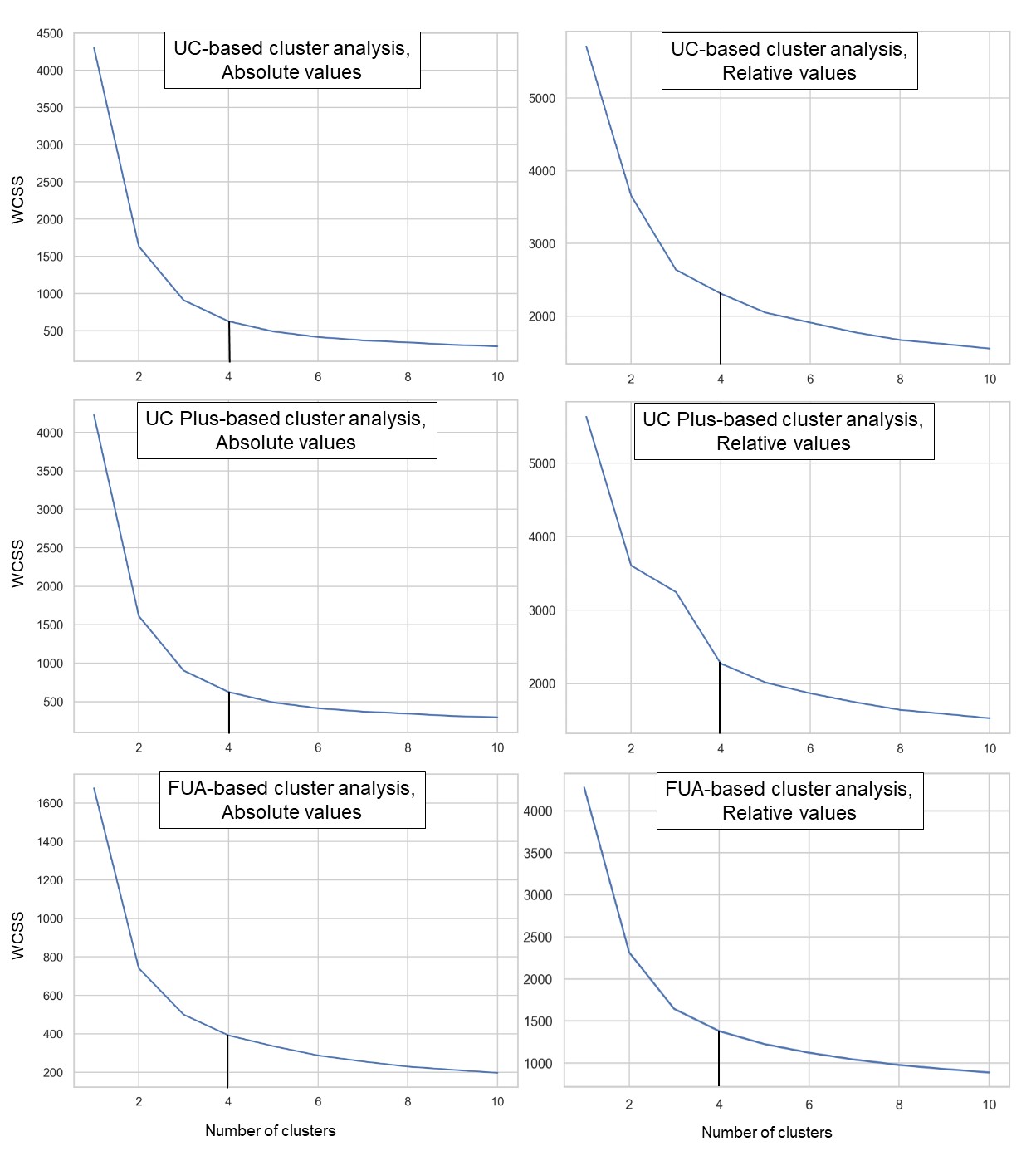


Figure S. 13. Results of the Elbow analysis to find the optimal number of cluster using the time-series k-means clustering algorithm for different city definitions and absolute vs. normalized time series. The “elbow” is considered to be located at the number of clusters (k) where the within-cluster sum of squares (WCSS) starts to decrease linearly with k.


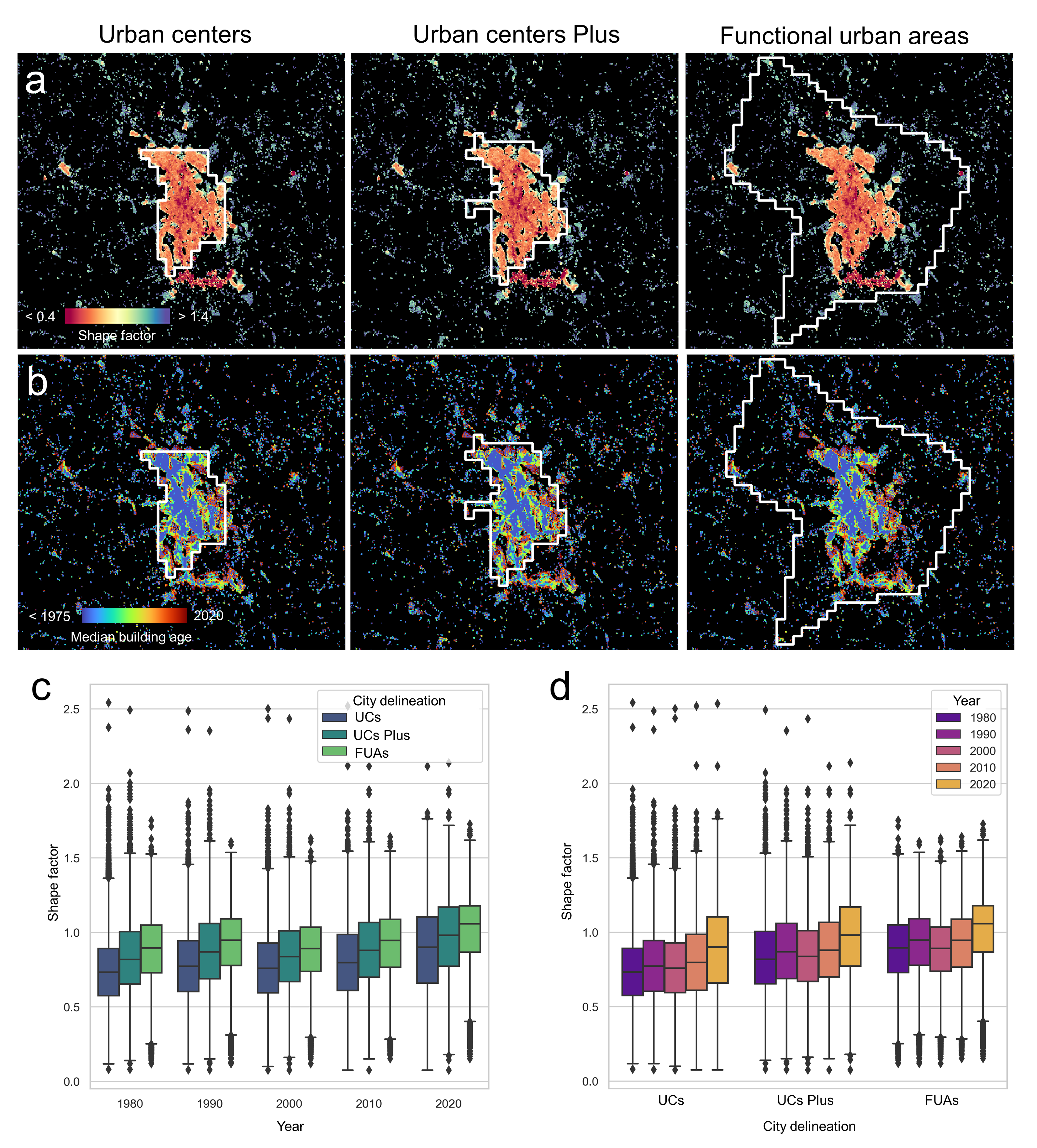


Figure S. 14. Different delineations of “cities” evaluated in this analysis: Urban centers from the Degree of Urbanization settlement model, “Urban Centers Plus”, encompassing urban centers plus adjacent peri-urban, sub-urban and semi-dense areas, and functional urban areas (FUAs), overlaid with (a) gridded average building compactness data derived from GHS-OBAT, and (b) GHS-AGE estimated median age of the built stock, both used as input for cluster analysis. The effect of the choice of city delineation method becomes evident when comparing the average building compactness distributions of the 100-m grid cell averages across all cities by building age group: The more peri-urban, sub-urban, semi-dense and/or rural areas are included in the city delineation, the higher the average shape factors (c). Consistent with findings in the main article, all three urban delineation methods yield increasing average building shape factors from old towards recent building age groups (d). Maps in (a) and (b) are shown for the city of Anápolis (Goiás, Brazil). Maps created with Matplotlib.

Table S. 3. Completeness statistics of GHS-OBAT for different city representations (“Urban Areas” - UAs) used in this analysis. The incomplete coverage of the GHS-OBAT building footprints is quantified by from the binarization of the gridded, average compactness data and GHS-AGE into covered vs. non-covered grid cells. This allows for calculating the intersection-over-union (IoU) of these coverages per UC. Assuming that GHS-AGE has a nearly-complete coverage of urban areas, low IoU values indicate lower levels of GHS-OBAT completeness. To avoid bias in the cluster analysis due to low coverage, UCs with an IoU of < 0.8 are discarded.

| Scenario | Urban areas (UAs) | UAs w IoU > 0.8 | UAs w IoU<0.8 | completeness % |
| --- | --- | --- | --- | --- |
| Urban Centres | 11543 | 9552 | 1982 | 82.81 |
| FUAs | 9031 | 6529 | 2502 | 72.29 |


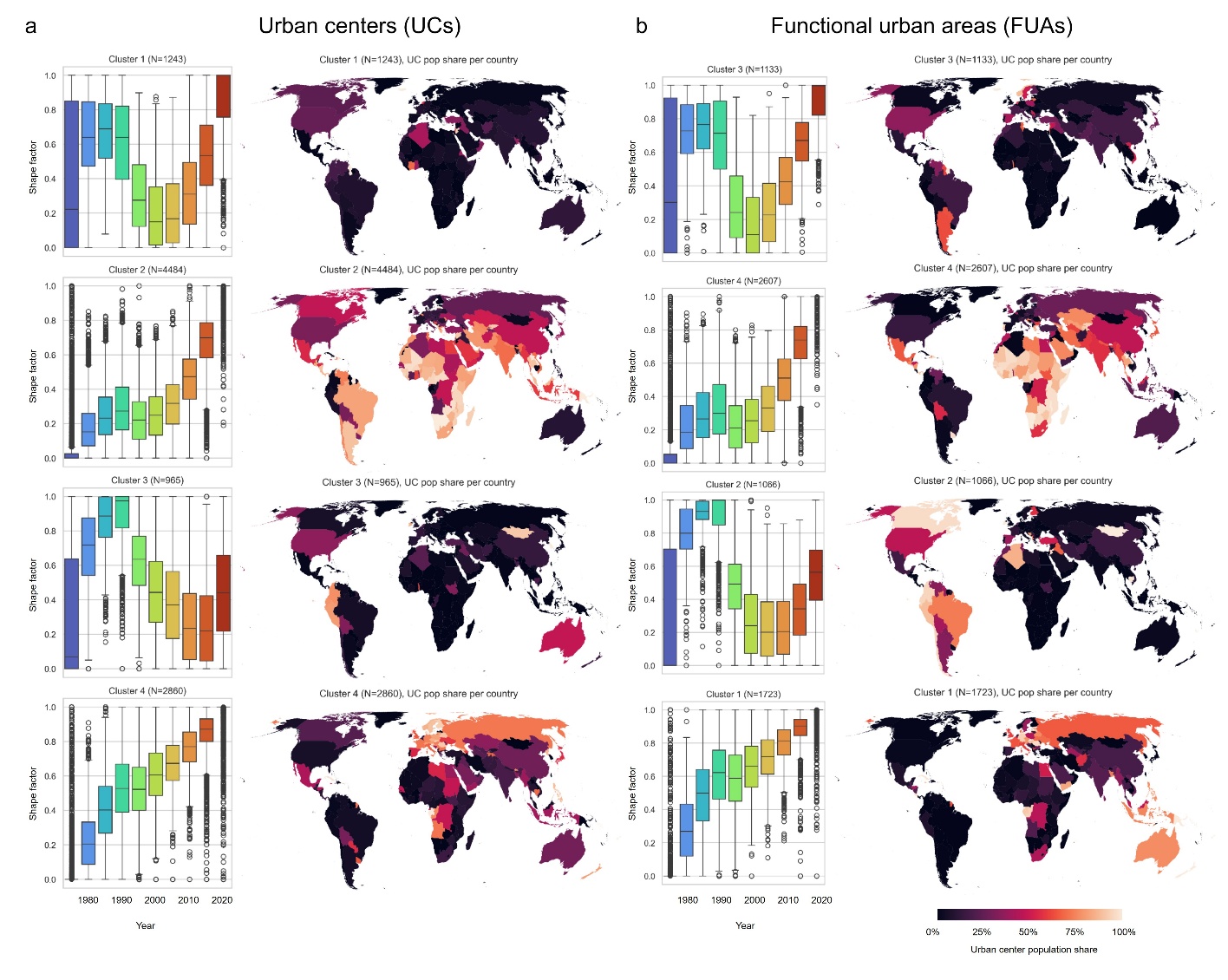


Figure S. 15. Time-series k-means results for normalized building compactness-age relationships for two different spatial delineations of cities: Urban centers (UCs) nd functional urban areas (FUAs). Both city delineations yield four clusters of relative (i.e., normalized) building compactness time series, exhibiting highly similar building compactness trends over time; these clusters show highly similar geospatial patterns regardness the choice of city delineation method. An exception are countries in North America, as well as Brazil, when using FUAs to delineate cities, resulting in a notable cluster switch. This is likely an effect of the larger geographic extents of FUAs, encompassing commuting-based urban influence zones. Due to the high development level of road infrastructure, along with vast peri-urban settlement structures in North America, FUAs can be very large as compared to other countries, and thus may capture more peri-urban and even rural areas, resulting in different average building compactness time series that yield different clustering results. Maps created with Matplotlib, with free vector and raster basemap data from Natural Earth @ naturalearthdata.com.


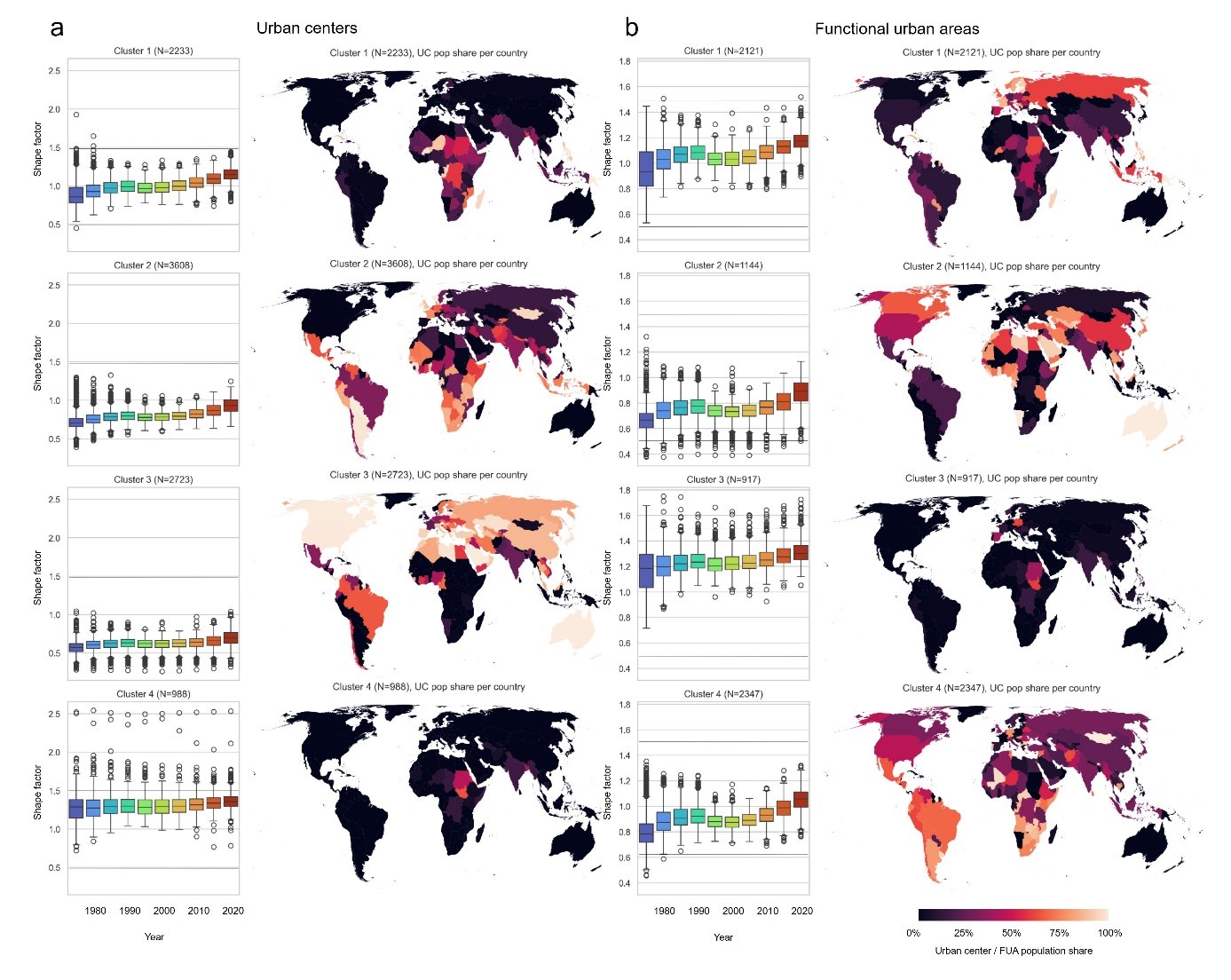


Figure S. 16. K-means cluster analysis results for building compactness time series associated with (a) urban centers and (b) functional urban areas, including their geographic distributions, when using non-normalized time series of building compactness instead of relative, normalized values. The resulting clusters indicate slightly increasing shape factors over time, but at different overall levels, reflecting the overall finding of higher average shape factors in low-income countries, as discussed in the main article. Differences between the results for urban centers and functional urban areas arise from the different underlying modelling strategies and resulting differences in geographic scope, with functional urban areas including much more peri-urban and even low-density rural areas located in the influence area (i.e., commuting zone) of urban centers. Maps created with Matplotlib, with free vector and raster basemap data from Natural Earth @ naturalearthdata.com.

1. <https://docs.overturemaps.org/guides/buildings/> [↑](#footnote-ref-2)
2. Beck, H. E. *et al.* High-resolution (1 km) Köppen-Geiger maps for 1901–2099 based on constrained CMIP6 projections. *Sci Data* **10**, 724 (2023). [↑](#footnote-ref-3)
3. <https://www.worldbank.org/en/building-green> [↑](#footnote-ref-4)
4. <https://www.iea.org/reports/renovation-of-near-20-of-existing-building-stock-to-zero-carbon-ready-by-2030-is-ambitious-but-necessary> [↑](#footnote-ref-5)
5. <https://build-up.ec.europa.eu/en/resources-and-tools/publications/deep-renovation-shifting-exception-standard-practice-eu> [↑](#footnote-ref-6)
